# Impact of Adaptation Currents on Synchronization of Coupled Exponential Integrate-and-Fire Neurons

**DOI:** 10.1371/journal.pcbi.1002478

**Published:** 2012-04-12

**Authors:** Josef Ladenbauer, Moritz Augustin, LieJune Shiau, Klaus Obermayer

**Affiliations:** 1 Department of Software Engineering and Theoretical Computer Science, Technische Universität Berlin, Berlin, Germany; 2 Bernstein Center for Computational Neuroscience Berlin, Berlin, Germany; 3 Department of Mathematics, University of Houston, Houston, Texas, United States of America; Indiana University, United States of America

## Abstract

The ability of spiking neurons to synchronize their activity in a network depends on the response behavior of these neurons as quantified by the phase response curve (PRC) and on coupling properties. The PRC characterizes the effects of transient inputs on spike timing and can be measured experimentally. Here we use the adaptive exponential integrate-and-fire (aEIF) neuron model to determine how subthreshold and spike-triggered slow adaptation currents shape the PRC. Based on that, we predict how synchrony and phase locked states of coupled neurons change in presence of synaptic delays and unequal coupling strengths. We find that increased subthreshold adaptation currents cause a transition of the PRC from only phase advances to phase advances and delays in response to excitatory perturbations. Increased spike-triggered adaptation currents on the other hand predominantly skew the PRC to the right. Both adaptation induced changes of the PRC are modulated by spike frequency, being more prominent at lower frequencies. Applying phase reduction theory, we show that subthreshold adaptation stabilizes synchrony for pairs of coupled excitatory neurons, while spike-triggered adaptation causes locking with a small phase difference, as long as synaptic heterogeneities are negligible. For inhibitory pairs synchrony is stable and robust against conduction delays, and adaptation can mediate bistability of in-phase and anti-phase locking. We further demonstrate that stable synchrony and bistable in/anti-phase locking of pairs carry over to synchronization and clustering of larger networks. The effects of adaptation in aEIF neurons on PRCs and network dynamics qualitatively reflect those of biophysical adaptation currents in detailed Hodgkin-Huxley-based neurons, which underscores the utility of the aEIF model for investigating the dynamical behavior of networks. Our results suggest neuronal spike frequency adaptation as a mechanism synchronizing low frequency oscillations in local excitatory networks, but indicate that inhibition rather than excitation generates coherent rhythms at higher frequencies.

## Author Summary

Synchronization of neuronal spiking in the brain is related to cognitive functions, such as perception, attention, and memory. It is therefore important to determine which properties of neurons influence their collective behavior in a network and to understand how. A prominent feature of many cortical neurons is spike frequency adaptation, which is caused by slow transmembrane currents. We investigated how these adaptation currents affect the synchronization tendency of coupled model neurons. Using the efficient adaptive exponential integrate-and-fire (aEIF) model and a biophysically detailed neuron model for validation, we found that increased adaptation currents promote synchronization of coupled excitatory neurons at lower spike frequencies, as long as the conduction delays between the neurons are negligible. Inhibitory neurons on the other hand synchronize in presence of conduction delays, with or without adaptation currents. Our results emphasize the utility of the aEIF model for computational studies of neuronal network dynamics. We conclude that adaptation currents provide a mechanism to generate low frequency oscillations in local populations of excitatory neurons, while faster rhythms seem to be caused by inhibition rather than excitation.

## Introduction

Synchronized oscillating neural activity has been shown to be involved in a variety of cognitive functions [1], [2] such as multisensory integration [3], [4], conscious perception [5],[6], selective attention [7], and memory [9], [10], as well as in pathological states including Parkinson's disease [11], schizophrenia [12], and epilepsy [13]. These observations have led to a great interest in understanding the mechanisms of neuronal synchronization, how synchronous oscillations are initiated, maintained, and destabilized.

The phase response curve (PRC) provides a powerful tool to study neuronal synchronization [14]. The PRC is an experimentally obtainable measure that characterizes the effects of transient inputs to a periodically spiking neuron on the timing of its subsequent spike. PRC based techniques have been applied widely to analyze rhythms of neuronal populations and have yielded valuable insights into, for example, motor pattern generation [15], the hippocampal theta rhythm [16], and memory retrieval [10]. The shape of the PRC is strongly affected by ionic currents that mediate spike frequency adaptation (SFA) [17], [18], a prominent feature of neuronal dynamics shown by a decrease in instantaneous spike rate during a sustained current injection [19]–[21]. These adaptation currents modify the PRC in distinct ways, depending on whether they operate near rest or during the spike [18]. Using biophysical neuron models, it has been shown that a low threshold outward current, such as the muscarinic voltage-dependent 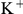-current (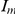), can produce a type II PRC, characterized by phase advances and delays in response to excitatory stimuli, in contrast to only phase advances, defining a type I PRC. A high threshold outward current on the other hand, such as the 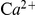-dependent afterhyperpolarization 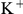-current (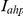), flattens the PRC at early phases and skews its peak towards the end of the period [18], [22], [23]. Both changes of the PRC indicate an increased propensity for synchronization of coupled excitatory cells [22], and can be controlled selectively through cholinergic neuromodulation. In particular, 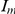 and 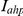 are reduced by acetylcholine with different sensitivities, which modifies the PRC shape [23]–[25].

In recent years substantial efforts have been exerted to develop single neuron models of reduced complexity that can reproduce a large repertoire of observed neuronal behavior, while being computationally less demanding and, more importantly, easier to understand and analyze than detailed biophysical models. Two-dimensional variants of the leaky integrate-and-fire neuron model have been proposed which take into consideration an adaptation mechanism that is spike triggered [26] or subthreshold, capturing resonance properties [27], as well as an improved description of spike initiation by an exponential term [28]. A popular example is the adaptive exponential leaky integrate-and-fire (aEIF) model by Brette and Gerstner [29], [30]. The aEIF model is similar to the two-variable model of Izhikevich [31], such that both models include a sub-threshold as well as a spike-triggered adaptation component in one adaptation current. The advantages of the aEIF model, as opposed to the Izhikevich model, are the exponential description of spike initiation instead of a quadratic nonlinearity, and more importantly, that its parameters are of physiological relevance. Despite their simplicity, these two models (aEIF and Izhikevich) can capture a broad range of neuronal dynamics [32]–[34] which renders them appropriate for application in large-scale network models [35], [36]. Furthermore, the aEIF model has been successfully fit to Hodgkin-Huxley-type neurons as well as to recordings from cortical neurons [29], [37], [38]. Since lately, this model is also implemented in neuromorphic hardware systems [39].

Because of subthreshold and spike-triggered contributions to the adaptation current, the aEIF model exhibits a rich dynamical structure [33], and can be tuned to reproduce the behavior of all major classes of neurons, as defined electrophysiologically in vitro [34]. Here, we use the aEIF model to study the influence of adaptation on network dynamics, particularly synchronization and phase locking, taking into account conduction delays and unequal synaptic strengths. First, we show how both subthreshold and spike-triggered adaptation affect the PRC as a function of spike frequency. Then, we apply phase reduction theory, assuming weak coupling, to explain how the changes in phase response behavior determine phase locking of neuronal pairs, considering conduction delays and heterogeneous synaptic strengths. We next present numerical simulations of networks which support the findings from our analysis of phase locking in neuronal pairs, and show their robustness against heterogeneities. Finally, to validate the biophysical implication of the adaptation parameters in the aEIF model, we relate and compare the results using this model to the effects of 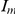 and 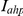 on synchronization in Hodgkin-Huxley-type conductance based neurons. Thereby, we demonstrate that the basic description of an adaptation current in the low-dimensional aEIF model suffices to capture the characteristic changes of PRCs, and consequently the effects on phase locking and network behavior, mediated by biophysical adaptation currents in a complex neuron model. The aEIF model thus represents a useful and efficient tool to examine the dynamical behavior of neuronal networks.

## Methods

### aEIF neuron model

The aEIF model consists of two differential equations and a reset condition,
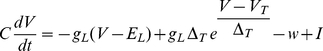
(1)

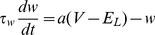
(2)

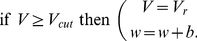
(3)The first equation (1) is the membrane equation, where the capacitive current through the membrane with capacitance 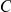 equals the sum of ionic currents, the adaptation current 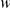, and the input current 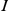. The ionic currents are given by an ohmic leak current, determined by the leak conductance 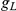 and the leak reversal potential 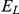, and a 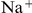-current which is responsible for the generation of spikes. The 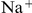-current is approximated by the exponential term, where 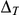 is the threshold slope factor and 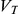 is the threshold potential, assuming that the activation of 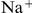-channels is instantaneous and neglecting their inactivation [28]. The membrane time constant is 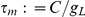. When 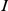 drives the membrane potential 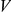 beyond 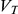, the exponential term actuates a positive feedback and leads to a spike, which is said to occur at the time when 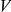 diverges towards infinity. In practice, integration of the model equations is stopped when 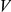 reaches a finite “cutoff” value 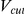, and 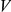 is reset to 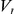 (3). Equation (2) governs the dynamics of 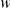, with the adaptation time constant 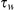. 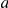 quantifies a conductance that mediates subthreshold adaptation. Spike-triggered adaptation is included through the increment 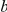 (3).

The dynamics of the model relevant to our study is outlined as follows. When the input current 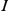 to the neuron at rest is slowly increased, at some critical current the resting state is destabilized which leads to repetitive spiking for large regions in parameter space [34]. This onset of spiking corresponds to a saddle-node (SN) bifurcation if 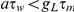, and a subcritical Andronov-Hopf (AH) bifurcation if 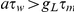 at current values 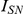 and 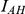 respectively which can be calculated explicitly [33]. In the former case a stable fixed point (the neuronal resting state) and an unstable fixed point (the saddle) merge and disappear, in the latter case the stable fixed point becomes unstable before merging with the saddle. In the limiting case 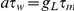, both bifurcations (SN and AH) meet and the system undergoes a Bogdanov-Takens (BT) bifurcation. The sets of points with 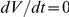 and 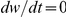 are called 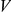-nullcline and 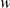-nullcline, respectively. It is obvious that all fixed points in the two-dimensional state space can be identified as intersections of these two nullclines. Spiking can occur at a constant input current lower than 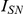 or 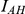 depending on whether the sequence of reset points lies exterior to the basin of attraction of the stable fixed point. This means, the system just below the bifurcation current can be bistable; periodic spiking and constant membrane potential are possible at the same input current. Thus, periodic spiking trajectories do not necessarily emerge from a SN or AH bifurcation. We determined the lowest input current that produces repetitive spiking (the rheobase current, 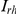) numerically by delivering long-lasting rectangular current pulses to the model neurons at rest. Note that in general 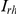 depends on 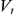, such that in case of bistability, 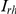 can be reduced by decreasing 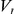 [33].

**Figure 1:**
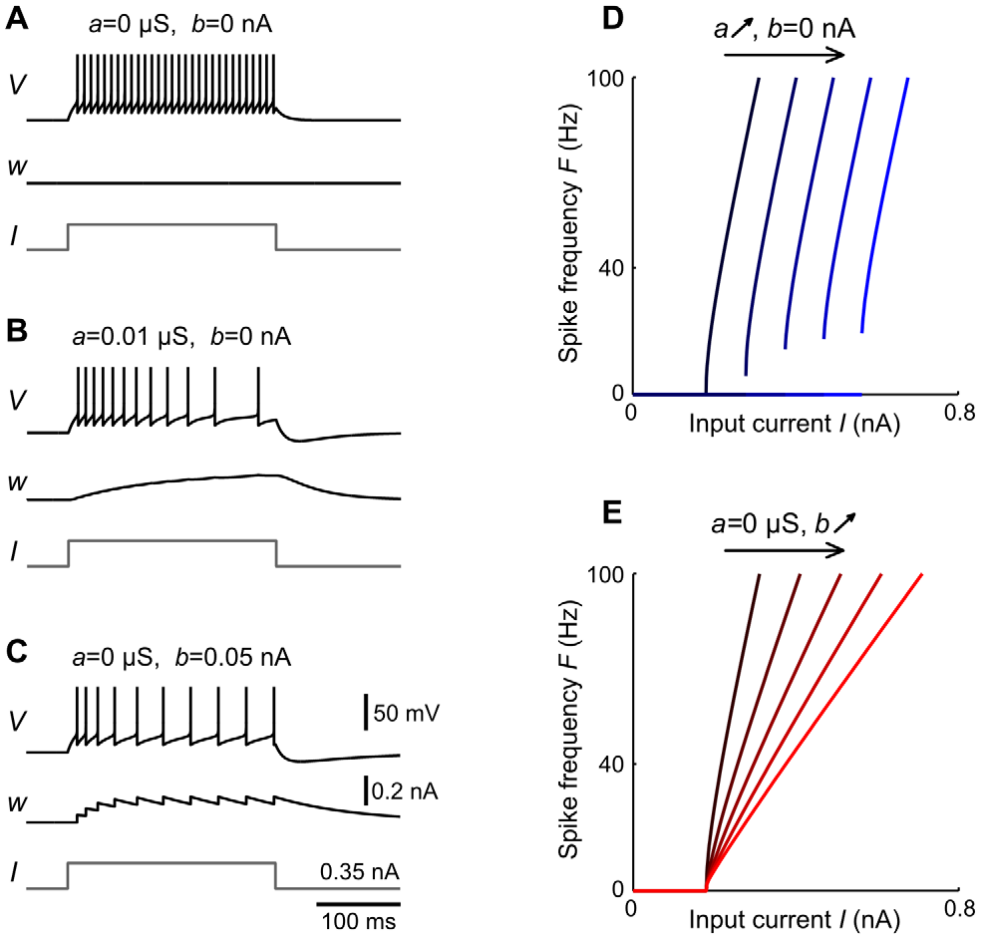
Influence of adaptation on spiking behavior and 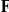-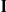 curves of aEIF neurons. A–C: Membrane potential 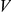 and adaptation current 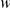 of aEIF neurons without adaptation (A), with subthreshold adaptation (B) and with spike-triggered adaptation (C), in response to step currents 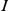. To demonstrate the steep increase of 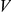 past 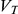, 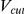 was set to 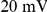. Note that the neuron in C has not reached its steady state frequency by the end of the rectangular current pulse. D,E: 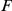-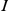 relationships for 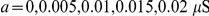, 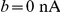 (black – blue, D) and 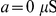, 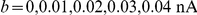 (black – red, E). All other model parameters used for this figure are provided in the Methods section.

We selected realistic values for the model parameters (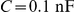, 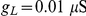, 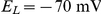, 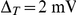, 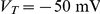, 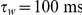, 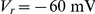) and varied the adaptation parameters within reasonable ranges (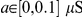, 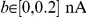). All model parametrizations in this study lead to periodic spiking for sufficiently large 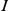, possibly including transient adaptation. Parameter regions which lead to bursting and irregular spiking [34] are not considered in this study. 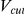 was set to 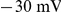, since from this value, even without an input current, 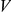 would rise to a typical peak value of the action potential (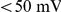) within less than 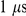 while 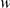 essentially does not change due to its large time constant. Only in Fig. 1A–C we used 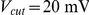 to demonstrate the steep increase of 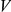 past 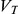.

### Traub neuron model

In order to compare the effects of adaptation in the aEIF model with those of 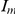 and 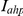 in a biophysically detailed model and with previously published results [18], we used a variant of the conductance based neuron model described by Traub et al. [41]. The current-balance equation of this model is given by
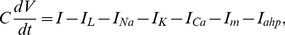
(4)where the ionic currents consist of a leak current 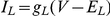, a 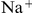-current 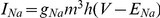, a delayed rectifying 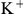-current 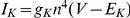, a high-threshold 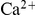-current 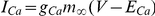 with 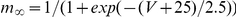, and the slow 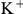-currents 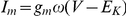, and 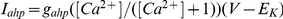. The gating variables 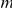, 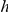 and 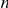 satisfy first-order kinetics
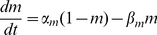
(5)

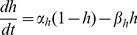
(6)

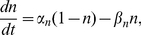
(7)with 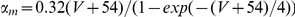 and 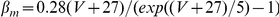, 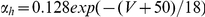 and 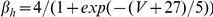, 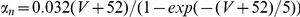 and 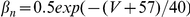. The fraction 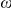 of open 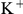-channels is governed by
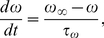
(8)where 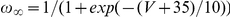, 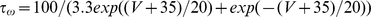, and the intracellular 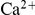 concentration 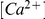 is described by
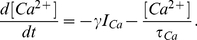
(9)Units are mV for the membrane potential and ms for time. Note that the state space of the Traub model eqs. (4)–(9) is six-dimensional.

The dynamics of interest is described below. Starting from a resting state, as 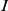 is increased, the model goes to repetitive spiking. Depending on the level of 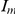, this (rest-spiking) transition occurs through a SN bifurcation for low values of 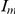 or a subcritical AH bifurcation for high values of 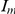, at input currents 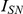 and 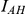, respectively. The SN bifurcation gives rise to a branch of stable periodic solutions (limit cycles) with arbitrarily low frequency. Larger values of 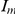 cause the stable fixed point to lose its stability by an AH bifurcation (at 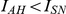). In this case, a branch of unstable periodic orbits emerges, which collides with a branch of stable limit cycles with finite frequency in a fold limit cycle bifurcation at current 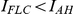. The branch of stable periodic spiking trajectories extends for currents larger than 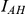 and 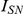. This means that in the AH bifurcation regime, the model exhibits hysteresis. That is, for an input current between 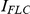 and 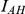 a stable equilibrium point and a stable limit cycle coexist. On the contrary, 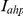 does not affect the bifurcation of the equilibria, since it is essentially nonexistent at rest.

We used parameter values as in [22]. Assuming a cell surface area of 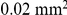, the membrane capacitance was 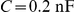, the conductances (in 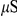) were 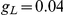, 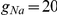, 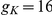, 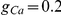, 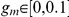, 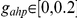, and the reversal potentials (in mV) were 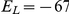, 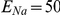, 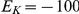, 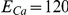; 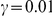 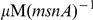 and 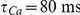.

### Network simulations

We considered networks of 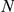 coupled neurons with identical properties using both models (aEIF and Traub), driven to repetitive spiking with period 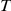,
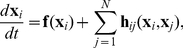
(10)where the vector 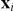 consists of the state variables of neuron 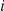 (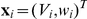 for the aEIF model, or 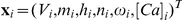 for the Traub model), 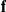 governs the dynamics of the uncoupled neuron (according to either neuron model) and the coupling function 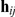 contains the synaptic current 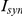 (received by postsynaptic neuron 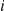 from presynaptic neuron 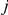) in the first component and all other components are zero. 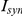 was modeled using a bi-exponential description of the synaptic conductance,
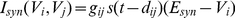
(11)

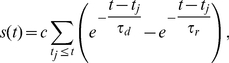
(12)where 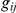 denotes the peak conductance, 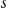 the fraction of open ion channels, 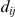 the conduction delay which includes axonal as well as dendritic contributions, and 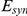 the synaptic reversal potential. 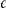 is a normalization factor which was chosen such that the peak of 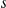 equals one. The spike times 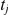 of neuron 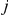 (at the soma) correspond to the times at which the membrane potential reaches 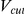 (in the aEIF model) or the peak of the action potential (in the Traub model). 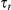 and 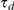 are the rise and decay time constants, respectively. For excitatory synapses the parameters were chosen to model an AMPA-mediated current (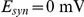, 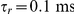, 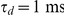), the parameters for inhbitory synapses we set to describe a 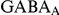-mediated current (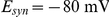, 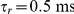, 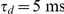).

We simulated the aEIF and Traub neuron networks, respectively, taking 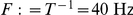, homogeneous all-to-all connectivity without self-feedback (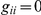), and neglecting conduction delays (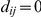). We further introduced heterogeneities of several degrees w.r.t. synaptic strengths and conduction delays to the computationally less demanding aEIF network. Specifically, 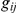 (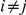) and 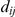 were sampled from a uniform distribution over various value ranges. The neurons were weakly coupled, in the sense that the total synaptic input received by a neuron from all other neurons in the network (assuming they spike synchronously) resulted in a maximal change of ISI (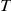) of less than 5%, which was determined by simulations. As initial conditions we used points of the spiking trajectory at times that were uniformly sampled from the interval 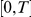, i.e. the initial states were asynchronous. Simulation time was 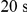 for each configuration of the aEIF networks and 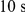 for the Traub neuron networks. All network simulations were done with BRIAN 1.3 [42] applying the second-order Runge-Kutta integration method with a time step of 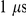 for coupled pairs and 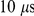 for larger networks.

We measured the degree of spike synchronization in the simulated networks using averaged pairwise cross-correlations between the neurons [43],
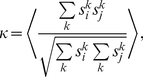
(13)where 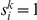 if neuron 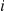 spikes in time interval 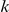, otherwise 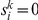, for 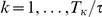. 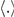 indicates the average over all neuronal pairs (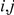) in the network. Calculation period 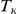 was 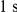 and time bin 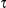 was 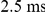. 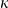 assumes a value of 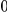 for asynchronous spiking and approaches 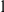 for perfect synchronization.

In order to quantify the degree of phase locking of neurons in the network we applied the mean phase coherence measure 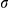 [44], [45] defined by
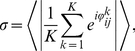
(14)where 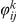 is the phase difference between neurons 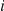 and 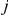 at the time of the 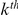 spike 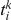 of neuron 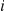, 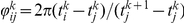. 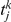 is the largest spike time of neuron 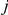 that precedes 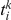, 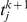 is the smallest spike time of neuron 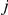 that succeeds 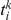. 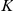 is the number of spikes of neuron 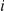 in the calculation period 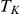. 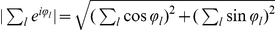 and 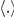 denotes the average over all pairs 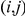. 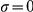 means no neuronal pair phase locks, 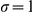 indicates complete phase locking. 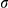 was calculated using for 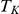 the last 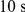 (aEIF networks) or 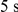 (Traub networks) of each simulation.

### PRC calculation

The PRC can be obtained (experimentally or in simulations) by delivering small perturbations to the membrane potential of a neuron oscillating with period 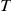 at different phases 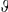 and calculating the change of the period. The PRC is then expressed as a function of phase as 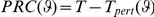, where 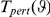 is the period of the neuron perturbed at 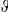. Positive (negative) values of 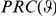 represent phase advances (delays). An alternative technique of determining the PRC is to solve the linearized adjoint equation [22], 
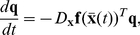
(15)subject to the normalization condition 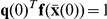 (see Text S1). 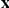, 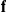 are as described above (cf. eq. (10)) and 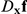 is the Jacobian matrix of 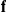. 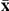 denotes the asymptotically stable 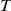-periodic spiking trajectory as a solution of the system
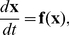
(16)of differential equations and a reset condition in case of the aEIF model. Eq. (16) together with the reset condition describe the dynamics of an uncoupled neuron. 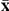 is an attractor of this dynamical system and nearby trajectories will converge to it. To obtain 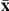, we integrated the neuron model equations for a given set of parameters and adjusted the input current 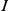, such that the period was 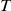. Analysis was restricted to the regular spiking regime (cf. [34] for the aEIF model). Parameter regions where bursting and chaotic spiking occurs were avoided.

For Traub model trajectories, the peak of the action potential is identified with phase 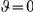, for aEIF trajectories 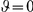 corresponds to the point of reset. The first component 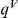 of the normalized 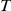-periodic solution 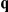 of eq. (15) represents the PRC, also called infinitesimal PRC, which characterizes the response of the oscillator to a vanishingly small perturbation (cf. Text S1). For continuous limit cycles 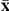, as produced by the Traub model, 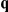 can be obtained by solving eq. (15) backward in time over several periods with arbitrary initial conditions. Since 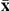 is asymptotically stable, the 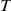-periodic solution of the adjoint system, eq. (15), is unstable. Thus, backward integration damps out the transients and we arrive at the periodic solution of eq. (15) [48]–[50]. In case of the aEIF model with an asymptotically stable 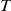-periodic solution 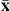, that involves a discontinuity in both variables 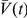, 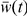 at integer multiples of 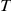, we treated the adjoint equations as a boundary value problem [18]. Specifically, we solved the adjoint system
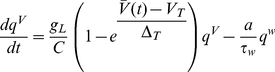
(17)

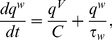
(18)subject to the conditions
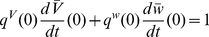
(19)

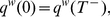
(20)where 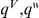 denote the two components of 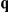, and 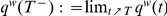 is the left-sided limit. Eq. (19) is the normalization condition. Eq. (20) is the continuity condition, which ensures 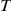-periodicity of the solution (see Text S1, derivation based on [51]–[53]). From the fact, that the end points of 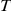-periodic aEIF trajectories differ, i.e. 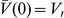, 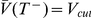 and 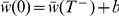, it follows that 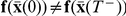, which in turn leads to 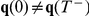. Perturbations of the same strength, which are applied to 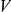 just before and after the spike, have therefore a different effect on the phase, leading to a discontinuity in the PRC.

The PRCs presented in this study were calculated using the adjoint method. For validation purposes, we also simulated a number of PRCs by directly applying small perturbations to the membrane potential 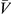 of the oscillating neuron at different phases and measuring the change in phase after many cycles – to ensure, that the perturbed trajectory had returned to the attractor 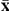. The results are in good agreement with the results of the adjoint method.

### Phase reduction

In the limit of weak synaptic interaction, which guarantees that a perturbed spiking trajectory remains close to the attracting (unperturbed) trajectory 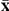, we can reduce the network model (10) to a lower dimensional network model where neuron 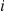 is described by its phase 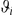 [48]–[50], [54], [55] as follows.
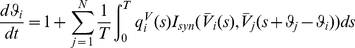
(21)

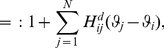
(22)where 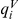 is the PRC of neuron 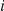 and 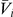 the first component (membrane potential) of the spiking trajectory 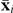 (see previous section and Text S1). 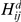 is the 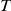-periodic averaged interaction function calculated using 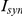 with conduction delay 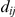 (11). Note that 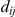 simply causes a shift in the interaction function: 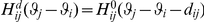. 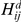 only depends on the difference of the phases (in the argument) which is a useful property when analyzing the stability of phase locked states of coupled neuronal pairs. In this case (without self-feedback as already assumed) the phase difference 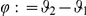 evolves according to the scalar differential equation
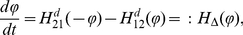
(23)whose stable fixed points are given by the zero crossings 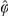 of 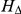 for which 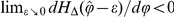 and 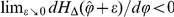. If 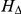 is differentiable at 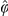, these left and right sided limits are equal and represent the slope. Note however that 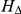 is continuous, but not necessarily differentiable due to the discontinuity of the PRC of an aEIF neuron. Therefore, the limits might not be equal in this case. The case where 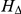 is discontinuous at 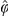, which can be caused by 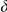-pulse coupling, i.e. 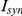 is replaced by a 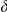-function, is addressed in the Results section. We calculated these stable fixed points, which correspond to stable phase locked states, for pairs of identical cells coupled with equal or heterogeneous synaptic strengths and symmetric conduction delays, 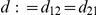, using PRCs derived from the aEIF and Traub neuron models, driven to 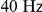 periodic spiking. Periodic spiking trajectories of both models and PRCs of Traub neurons were computed using variable order multistep integration methods, for PRCs of aEIF neurons a fifth-order collocation method was used to solve eqs. (17)–(20). These integration methods are implemented in MATLAB (2010a, The MathWorks). Bifurcation currents of the Traub model were calculated using MATCONT [56], [57].

## Results

### PRC characteristics of aEIF neurons

We first examine the effects of the adaptation components 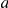 and 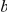, respectively, on spiking behavior of aEIF neurons at rest in response to (suprathreshold) current pulses (Fig. 1A–C). Without adaptation (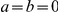) the model produces tonic spiking (Fig. 1A). Increasing 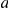 or 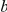 leads to SFA as shown by a gradual increase of the inter spike intervals (ISI) until a steady-state spike frequency 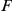 is reached. Adaptation current 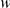 builds up and saturates slowly when only conductance 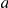 is considered (Fig. 1B) in comparison to spike-triggered increments 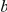 (Fig. 1C). Fig. 1D,E depicts the relationship between 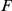 and the injected current 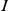 for various fixed values of 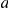 and 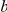. Increased subthreshold adaptation causes the minimum spike frequency to jump from zero to a positive value, producing a discontinuous 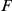-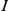 curve (Fig. 1D). A continuous (discontinuous) 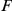-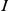 curve indicates class I (II) membrane excitability which is typical for a SN (AH) bifurcation at the onset of spiking respectively. An increase of 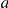 causes this bifurcation to switch from SN to AH, thereby changing the membrane excitability from class I to II, shown by the 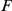-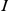 curves. An increase of 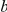 on the other hand does not produce a discontinuity in the 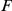-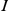 curve, i.e. the membrane excitability remains class I (Fig. 1E). Furthermore, increasing 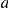 shifts the 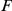-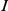 curve to larger current values without affecting its slope, while an increase of 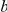 decreases the slope of the 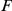-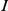 curve in a divisive manner. When 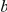 is large, the neuron is desensitized in the sense that spike frequency is much less affected by changes in the driving input.

**Figure 2:**
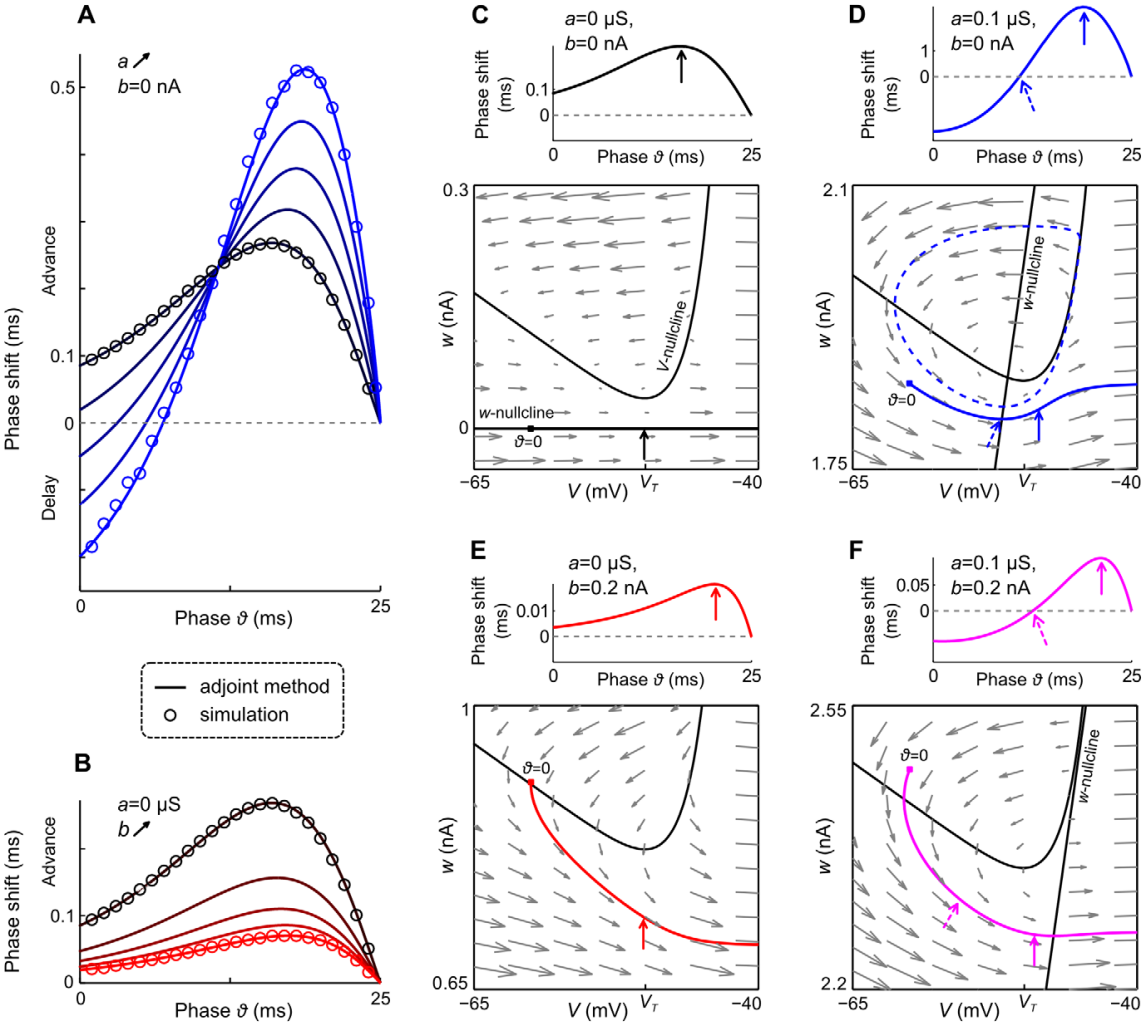
Effects of adaptation on PRCs of aEIF neurons. A,B: PRCs associated with adaptation parameters as in Fig. 1D,E. Solid curves are PRCs calculated with the adjoint method and scaled by 0.1 mV, circles denote PRC points that were obtained from numerical simulations of eqs. (1)–(3), using 0.1 mV perturbations at various phases 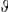 (see Methods and Text S1). The input currents 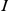 were chosen to ensure 40 Hz spiking. Note that the discontinuity of the PRCs at 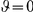 is caused by the reset of the spiking trajectories. C–F Top: PRCs for adaptation parameters as indicated and 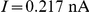 (C), 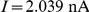 (D), 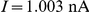 (E), 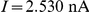 (F). C–F Bottom: Vector field, 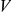
- and 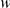-nullclines, and periodic spiking trajectory in the respective state space. The reset point (solid square) of the trajectory corresponds to the phase 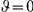. A solid arrow marks the location along the trajectory where the PRC (shown above) has its maximum. Dashed arrows in D, F mark the trajectory points that correspond to the zero crossings of the PRCs. Trajectory points change slowly in regions where the vector field magnitudes are small. The dashed blue curve in D denotes the boundary of the domain of attraction of the fixed point, which is located at the intersection of the nullclines. Note that differences in the vector fields and 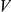-nullclines between C and E as well as D and F, are due to the changes in 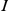.

In Fig. 2A,B we show how 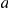 and 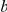 differentially affect the shape of the PRC of an aEIF neuron driven to periodic spiking. The PRCs calculated using the adjoint method (solid curves) match well with those obtained from simulations (circles). While non-adapting neurons have monophasic (type I) PRCs, which indicate only advancing effects of excitatory perturbations, increased levels of 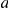 produce biphasic (type II) PRCs with larger magnitudes, which predict a delaying effect of excitatory perturbations received early in the oscillation cycle. An increase of 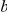 on the other hand flattens the PRC at early phases, shifts its peak towards the end of the period and reduces its magnitude. The type of the PRC however remains unchanged (type I). Indeed, if 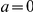 the PRC must be type I, since in this case the component 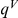 of the solution of the adjoint system, eqs. (17)–(20), can be written as 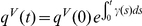, where 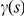 is given by the right-hand side of eq. (17). Thus, 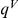 cannot switch sign.

To provide an intuitive explanation for the effects of adaptation on the PRC, we show the vector fields, 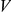
- and 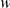-nullclines, and periodic spiking trajectories of four aEIF neurons (Fig. 2C–F). One neuron does not have an adaptation current (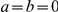), two neurons possess only one adaptation mechanism (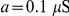, 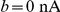 and 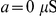, 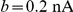, respectively) and for one both adaptation parameters are increased (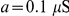, 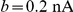). An excitatory perturbation to the non-adapting neuron at any point of its trajectory, i.e. at any phase, shifts this point closer to 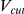 along the trajectory, which means the phase is shifted closer to 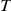, hence the advancing effect (Fig. 2C). The phase advance is strongest if the perturbing input is received at the position along the trajectory around which the vector field has the smallest magnitude, i.e. where the trajectory is “slowest”. In case of subthreshold adaptation (Fig. 2D), the adapted periodic spiking trajectory starts at a certain level of 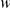 which decreases during the early part of the oscillation cycle and increases again during the late part, after the trajectory has passed the 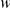-nullcline. A small transient excitatory input at an early phase pushes the respective point of the trajectory to the right (along the 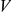-axis) causing the perturbed trajectory to pass through a region above the unperturbed trajectory, somewhat closer to the fixed point around which the vector field is almost null. Consequently, the neuron is slowed down and the subsequent spike delayed. An excitatory perturbation received at a later phase (to the right of the dashed arrow) causes phase advances, since the perturbed trajectory either remains nearly unchanged, however with a shorter path to the end of the cycle, compared to the unperturbed trajectory, or it passes below the unperturbed one where the magnitude of the vector field (pointing to the right) is larger. Note that for the parametrization in Fig. 2D, both, the resting state as well as the spiking trajectory are stable. In this case, a strong depolarizing input at an early phase can push the corresponding trajectory point into the domain of attraction of the fixed point, encircled by the dashed line in the figure, which would cause the resulting trajectory to spiral towards the fixed point and the neuron would stop spiking. On the other hand, increasing 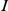 would shrink the domain of attraction of the fixed point and at 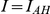, it would be destabilized by a subcritical AH bifurcation. When 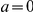 and 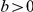, we obtain a type I PRC (Fig. 2E), as explained above. The advancing effect of an excitatory perturbation is strongest late in the oscillation cycle, indicated by the red arrow, where the perturbation pushes a trajectory point from a “slow” towards a “fast” region closer to the end of the cycle, as shown by the vector field. When 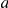 as well as 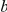 are increased, the PRC exhibits both adaptation mediated features (type II and skewness), see Fig. 2F. A push to the right along the corresponding trajectory experienced early in the cycle brings the perturbed trajectory closer to the fixed point and causes a delayed next spike. Such an effect persists even if the fixed point has disappeared due to a larger input current. In this case, the region where the fixed point used to be prior to the bifurcation, known as “ghost” of the fixed point, the vector field is still very small. This means that type II PRCs can exist for larger input currents 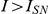. Note that differences of the vector fields and the shift of the nullclines relative to each other in Fig. 2C,D as well as Fig. 2E,F are due to different input current values (as an increase of 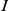 moves the 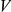-nullcline upwards). The maximal phase advances, indicated by solid arrows in Fig. 2A,B, are close to the threshold potential 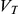 (where the 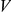-nullcline has its minimum) in all four cases.

**Figure 3:**
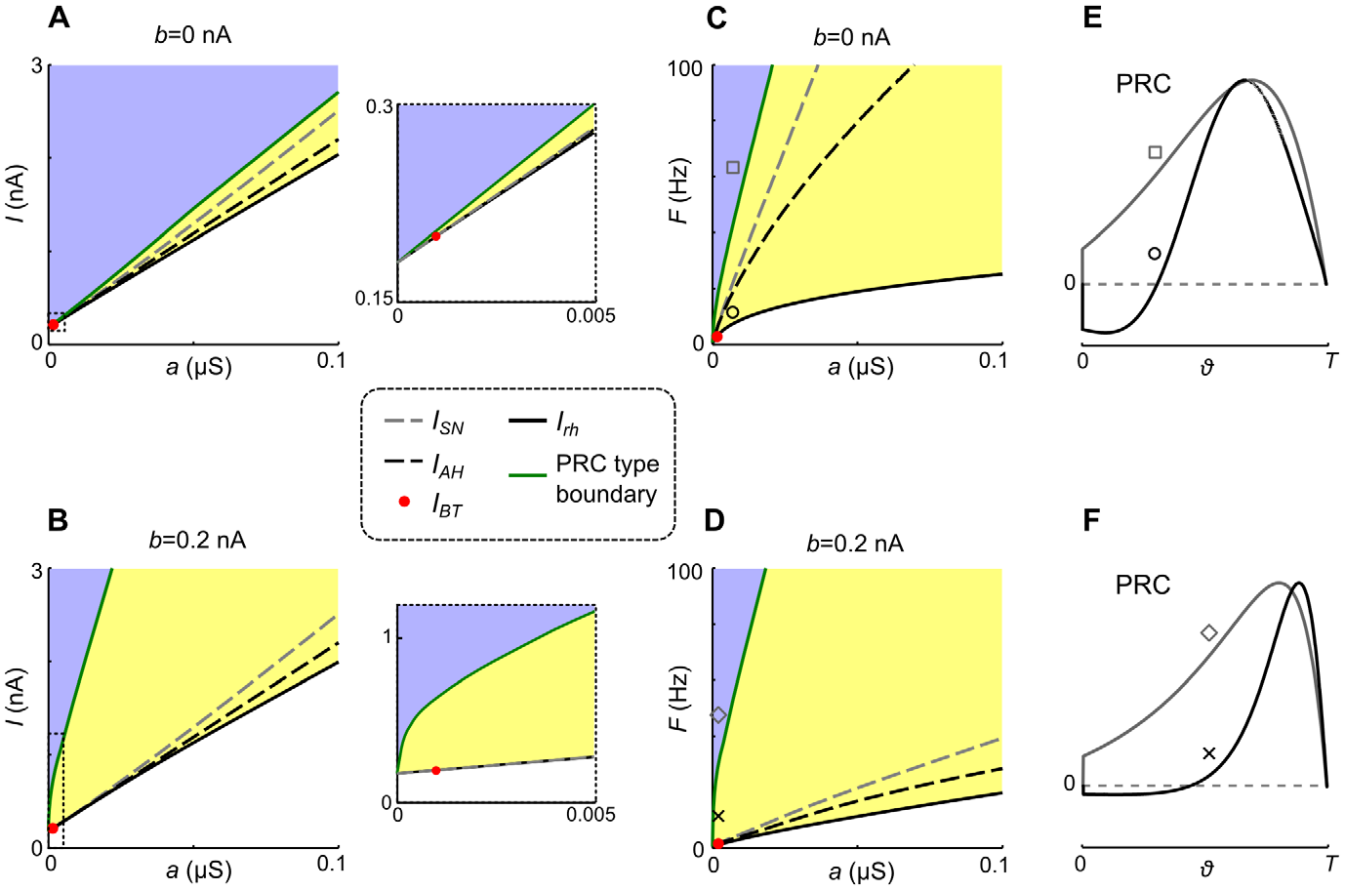
Bifurcation currents of the aEIF model and dependence of PRC characteristics on the input current. A,B: Rheobase current (solid black), SN and AH bifurcation currents 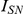, 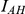 (dashed grey, dashed black) respectively, as well as input current (green) which separates type I (blue) and type II (yellow) PRC regions, as a function of 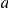, for 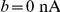 (A) and 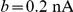 (B). At 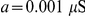 a BT bifurcation occurs at 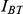 (where the SN and the AH bifurcations meet) marked by the red dot. The region around 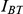 is displayed in a zoomed view. If 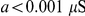 the system undergoes a SN bifurcation at 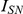, if 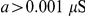 an AH bifurcation occurs at 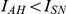. C,D: Spike frequencies 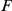 corresponding to the input currents in A and B. Note that the region in 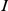-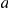 space where the PRCs are type II is very shallow in A compared to B, the corresponding regions in 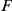-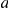 space shown in C and D however are rather similar. This is due to the steep (flat) 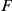-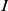 relationship for 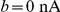 (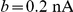) respectively (see Fig. 1D,E). E,F: PRCs with locations in 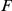-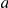 space as indicated, scaled to the same period 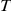.

We next investigate how the changes in PRCs caused by either adaptation component are affected by the spike frequency. Bifurcation currents, rheobase currents and corresponding frequencies, in dependence of 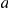 and 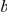, as well as regions in parameter space where PRCs are type I and II, are displayed in Fig. 3A–D. Fig. 3E,F shows how individual PRCs are modulated by spike frequency (input current). Both PRC characteristics, caused by 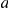 and 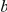, respectively, are more pronounced at low frequencies. Increasing 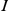 changes a type II PRC to type I and shifts its peak towards an earlier phase. The input current which separates type I and type II PRC regions (in parameter space) increases with both, 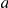 and 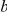 (Fig. 3A,B). That is, an increase of 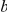 can also turn a type I into a type II PRC, by bringing the spiking trajectory closer to the fixed point or its “ghost”. This is however only possible if the system is in the AH bifurcation regime (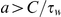) or close to it. Spike-triggered adaptation thereby considerably influences the range of input currents for which the PRCs are type II. The spike frequency according to the input current, at which a type II PRC turns into type I increases substantially with increasing 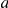, but only slighly with an increase of 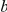 (Fig. 3C,D). The latter can be recognized by the similarity of the respective (green) curves in the subfigures C and D. Type II PRCs thus only exist in the lower frequency band whose width increases with increasing subthreshold adaptation.

### Phase locking of coupled aEIF pairs

In this section, we examine how the changes in phase response properties due to adaptation affects phase locking of coupled pairs of periodically spiking aEIF neurons. Specifically, we first analyze how the shape of the PRC determines the fixed points of eq. (23) and their stability, and then show how the modifications of the PRC mediated by the adaptation components 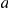 and 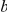 change those fixed points. Finally, we investigate the effects of conduction delays and heterogeneous coupling strengths on phase locking in dependence of adaptation.

#### Relation between phase locking and the PRC

In case of identical cell pairs and symmetric synaptic strengths, 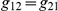, the interaction functions in eq. (23) are identical, 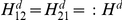, where 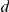 is the conduction delay. 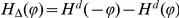 then becomes an odd, 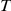-periodic function, which has roots at 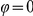 and 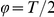. Thus, the in-phase and anti-phase locked states always exist. The stability of these two states can be “read off” the PRC even without having to calculate 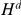, as is explained below. Let 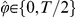 in the following. The fixed point 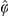 of eq. (23) is stable if 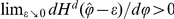 and 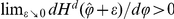. Note that the left and right sided limits are not equal if 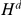 is not differentiable at 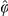, due to the discontinuity of the PRC of an aEIF neuron.

First, consider a synaptic current with infinitely fast rise and decay. In this case we use a positive (or negative) 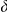-function in eq. (21) instead of 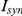 to describe the transient excitatory (or inhibitory) pulse. 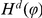 is then given by
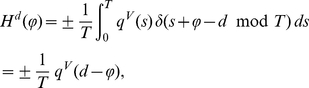
(24)that is, 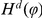 becomes the PRC, mirrored at 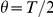, rightwards shifted by the delay 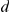 and scaled by 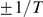. The sign of the slope of 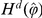 is thus given by the negative (positive) sign of the PRC slope at 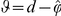, 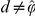, for excitatory (inhibitory) synapses respectively. For the aEIF model, the case 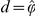 requires a distinction, because 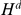 and 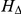 are discontinuous at 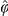. Let 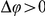 be the distance between 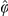 and the closest root of 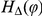. Since 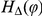 is odd and 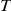-periodic, 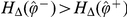 implies stability of 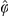, in the sense that 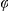 increases on the interval 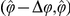 and decreases over 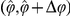. Thus, 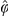 can be considered an attractor. 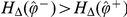 is equivalent to 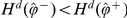 which in turn is equivalent to 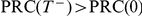 for excitatory coupling and 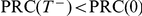 for inhibitory coupling. Hence, it is the discontinuity of the PRC which determines the stability of 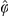 in this case.

**Figure 4:**
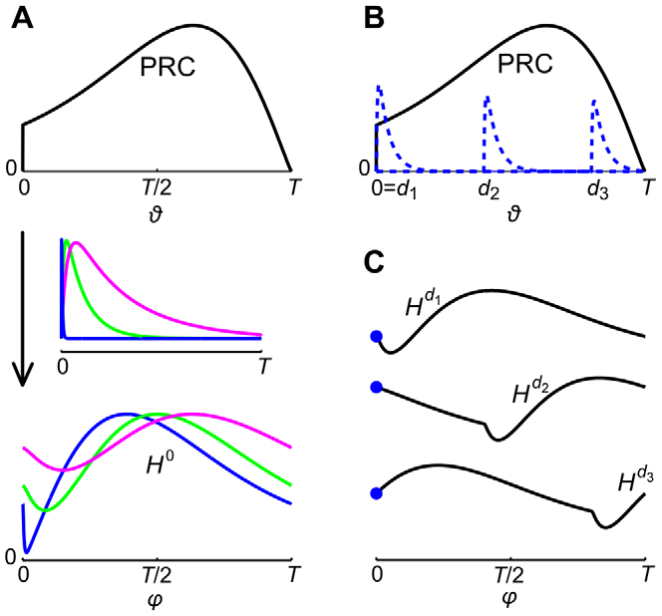
Relationship between the PRC and the interaction function. A: PRC of an aEIF neuron (top) spiking at 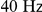 and interaction functions 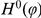 (bottom) obtained for synaptic conductances with three different sets of synaptic time constants: 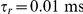, 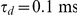 (blue), 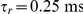, 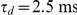 (green); 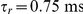, 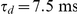 (magenta), and 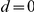. The synaptic current 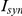 associated with each pair of time constants (center) illustrates the three synaptic timescales relative to the period 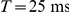. Note that 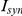 shown here is received by the neuron at the beginning of its ISI. B: PRC (solid black) of an aEIF neuron spiking at 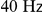 and excitatory synaptic currents 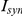 with 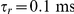, 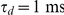 (dashed blue) received at three different phases. Assuming the input comes from a second, synchronous neuron, these phases represent three different conduction delays 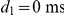, 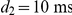, and 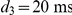. Note that synaptic input received at an earlier phase causes a larger peak of 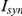, due to the smaller value 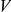 of the membrane potential which leads to a larger difference 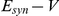 to the synapse's reversal potential 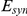. C: Interaction functions 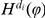 for pairs of neurons with the PRC shown in B, coupled by excitatory synapses with 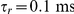, 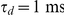, and delays 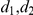 and 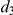. The values of 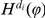 at 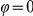 are highlighted by blue circles. The slopes of 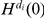, in terms of both left and right sided limits 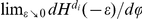 and 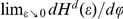, indicate whether the synchronous states are stable or unstable (see main text).

A synaptic current with finite rise and decay times causes an additional rightwards shift and a smoothing of the interaction function. The stability of the fixed point 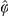 is then determined by the slope of the PRC and its discontinuity on the interval 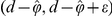, where 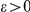 is on the order of the synaptic timescale. If the PRC slope is negative on this interval and its discontinuity (if occurring in the interval) is also negative, i.e. 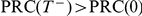, then 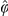 is stable for excitatory coupling and unstable for inhibitory coupling. In Fig. 4A we show the effect of the synaptic timescale, i.e. 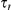 and 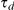, on the interaction function for a given PRC. Fig. 4B,C illustrates how the stability of the synchronous state of a neuronal pair is given by the slope of the PRC, for three different delays. The slope of the PRC is positive at 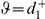, 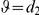 and negative at 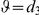 and remains positive (negative) until 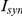 has decayed to a small value. Therefore, synchrony is unstable for delays 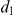, 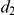 and stable for 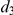, indicated by the slope of 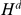 at 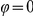, which is negative for the first two and positive for the third delay.

#### Effects of adaptation on phase locking of coupled aEIF pairs

First, consider pairs of identical aEIF neurons with the PRCs shown in Fig. 2A,B, symmetrically coupled through instantaneous synapses (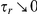 and 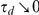) and without conduction delays (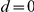). When the coupling is excitatory, the in-phase locked state (synchrony) is unstable in case of type I PRCs, since they have a positive “jump” at 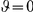, i.e. 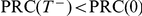. Synchrony is stable for pairs with type II PRCs however, as 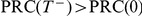. The anti-phase locked state on the other hand is unstable because of the positive PRC slopes at 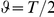. In case of inhibitory coupling, synchrony is stable for type I pairs and the anti-phase locked state is stable for all pairs. This means, bistability of in-phase and anti-phase locking occurs for inhibitory neurons with type I PRCs.

**Figure 5:**
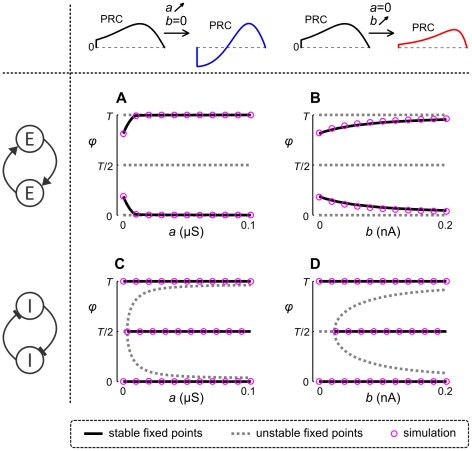
Effects of adaptation on phase locked states of coupled aEIF pairs. Stable (solid black) and unstable (dashed grey) phase locked states of pairs of aEIF neurons spiking at 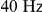 with identical PRCs as a function of adaptation parameters. These phase locked states were obtained by evaluating the interaction function. Circles denote the steady-state phase differences by numerically simulating pairs of aEIF neurons according to eqs. (1)–(3). To detect bistability, the simulations were run multiple times and the pairs initialized either near in-phase or anti-phase with values of the periodic spiking trajectory. In A and B the neurons are coupled through excitatory, in C and D through inhibitory synapses, as indicated by the diagrams on the left. Synaptic conductances are equal (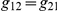) and conduction delays are not considered here (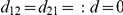). Synaptic time constants were 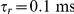, 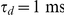 for excitatory and 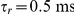, 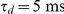 for inhibitory connections. In A and C, 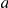 varies from 0 to 0.1 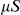 with 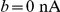, whereas in B and D, 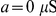 while 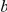 varies from 0 to 0.2 nA. All other model parameters are given in the Methods section. The corresponding changes in PRCs are indicated in the top row.

Next, we consider pairs that are coupled through synaptic currents 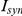 with finite rise and decay times, as described in the Methods section. In Fig. 5 we show how the stable (and unstable) phase locked states of pairs of neurons with symmetric excitatory (A, B) and inhibitory (C, D) synaptic interactions and without conduction delays change, when the PRCs are modified by the adaptation components 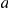 and 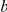. For excitatory pairs, stable fixed points shift towards synchrony, when 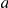 or 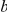 is increased. The phase differences become vanishinly small, when the PRCs switch from type I to type II due to subthreshold adaptation. Perfect synchrony is stabilized, where the PRC slopes at 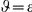 for small 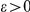 become negative, due to even larger values of 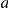 (not shown) or lower spike frequency (see Fig. 3C–F). Neurons that have type I PRCs with a pronounced skew, as caused by spike-triggered adaptation, lock almost but not completely in-phase, if the adaptation is sufficiently strong. Inhibitory pairs on the other hand show stable synchrony independent of PRC type and skewness. Larger values of 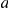 or 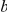 lead to additional stabilization of the anti-phase locked state. That is, strong adaptation in inhibitory pairs mediates bistability of in-phase and anti-phase locking. All phase locking predictions from the phase reduction approach are in good agreement with the results of numerically simulated coupled aEIF pairs.

#### Phase locking of aEIF pairs coupled with delays

**Figure 6:**
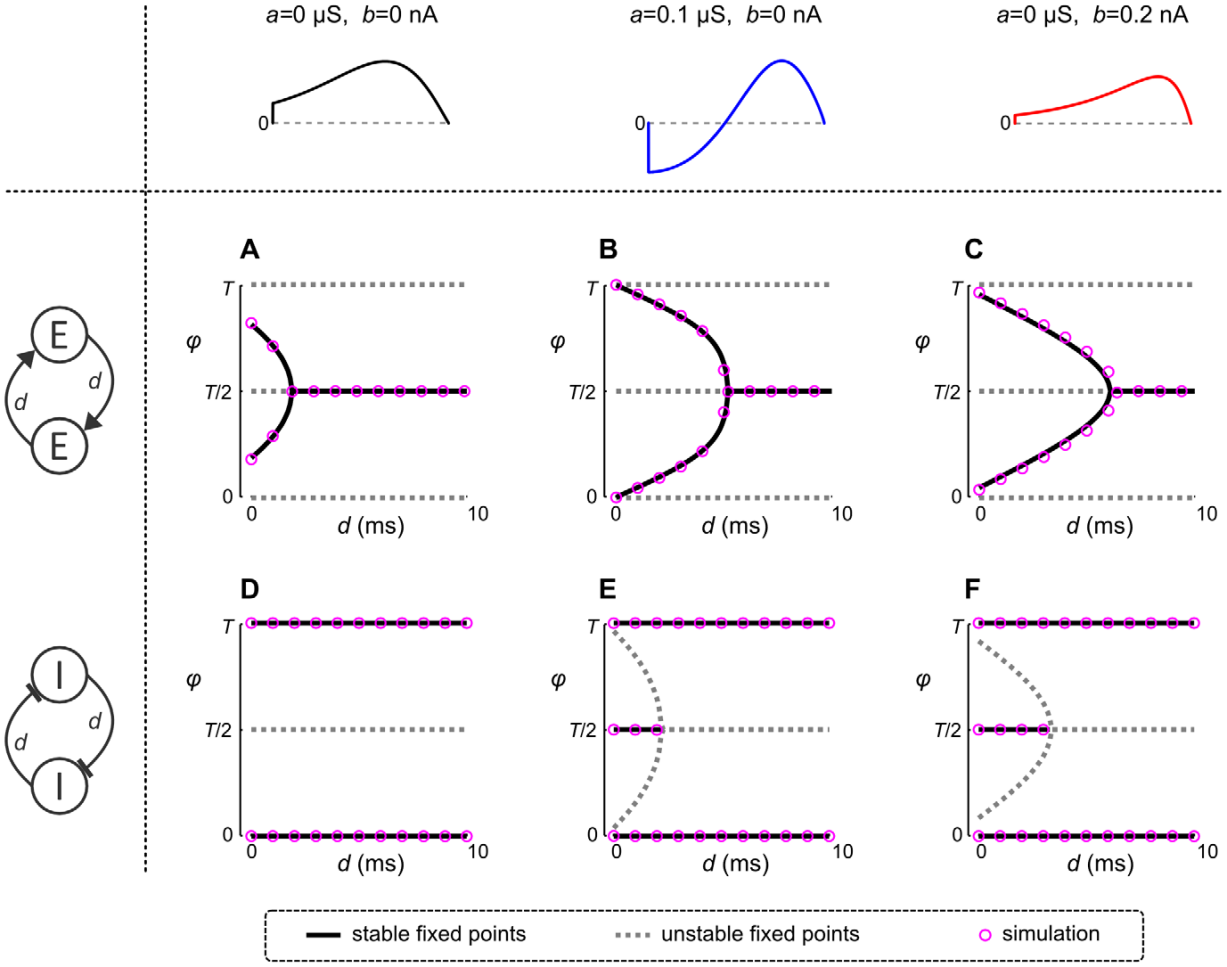
Phase locking of coupled aEIF pairs with conduction delays. Stable (solid black) and unstable (dashed grey) phase locked states of aEIF pairs without adaptation, 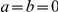 (A and D), and with adaptation, 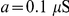, 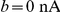 (B and E), 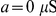, 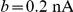 (C and F), as a function of the conduction delay 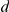. The neurons are coupled through excitatory (A–C) or inhibitory synapses (D–F) with equal conductances (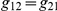). Synaptic time constants are as in Fig. 5. Circles denote steady-state phase differences of numerically simulated pairs of aEIF neurons. The corresponding PRCs are shown in the top row. 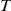 was 25 ms.

**Figure 7:**
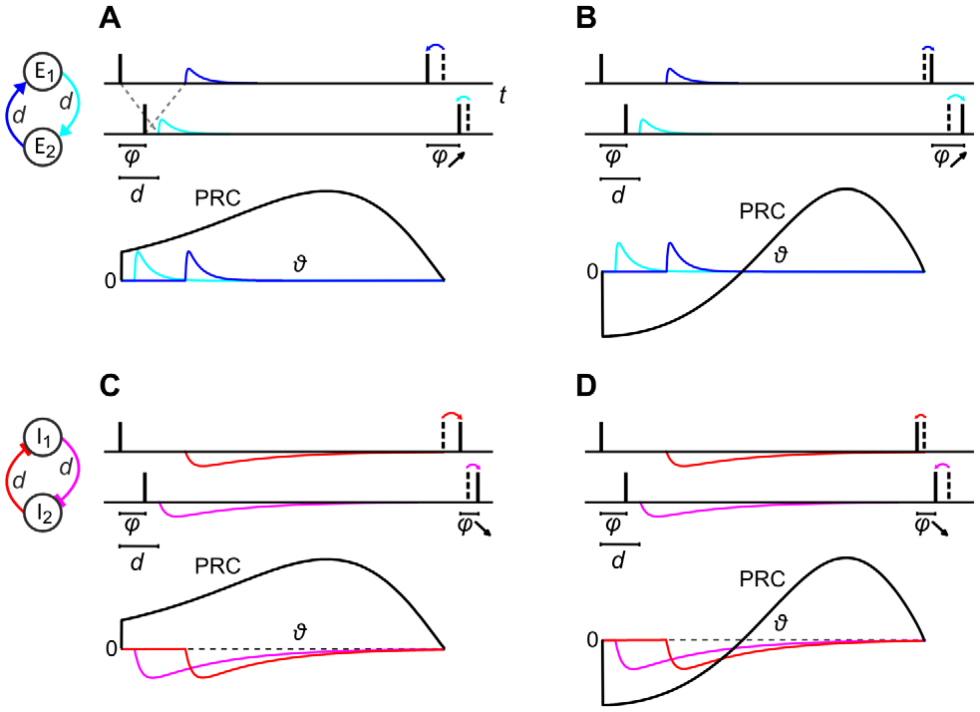
Effects of conduction delays on the stability of synchrony in coupled pairs. Spike times (solid bars) of two neurons oscillating with a small phase difference 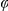 and coupled through excitatory (A and B) or inhibitory synapses (C and D) with a symmetric conduction delay 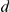. The PRCs of the neurons that make up each pair are displayed below. In A and C the neurons have type I PRCs, in B and D the PRCs are type II. The time (phase) at which each neuron receives a synaptic current is shown along the spike trace. Phase advances or delays, considering the time of input arrival and the shape of the PRC, are indicated by advanced or delayed subsequent spike times. Dashed bars indicate spike times without synaptic inputs. The consequent changes in 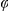 are highlighted.

We next investigate how phase locked states of excitatory and inhibitory pairs are affected by synaptic currents that involve conduction delays, considering the PRC of a neuron without adaptation, and two PRCs that represent adaptation induced by either 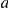 or 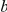. Neurons symmetrically coupled through excitatory synapses with a conduction delay do not synchronize irrespective of whether adaptation is present or not (Fig. 6A–C). Instead, stable states shift towards anti-phase locking with increasing mutual delays. Inhibitory pairs on the other hand synchronize for all conduction delays (Fig. 6D–F), but the anti-phase locked states of coupled inhibitory neurons with type II PRCs or skewed type I PRCs are destabilized by the delays. The bistable region is larger in case of spike-triggered adaptation compared to subthreshold adaptation (Fig. 6E,F). Again, all stable phase locked states obtained using phase reduction are verified by numerical simulations. Fig. 7 illustrates the phenomenon that synchronous spiking of excitatory pairs is destabilized by the delay, while synchrony remains stable for inhibitory pairs. Consider two neurons oscillating with a small phase difference 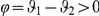 (neuron 1 slightly ahead of neuron 2). Then, a synaptic input received by neuron 2 at a delay 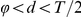 after neuron 1 has spiked, arrives at an earlier phase (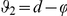) compared to the phase at which neuron 1 receives its input (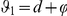). Consequently, if the synapses are excitatory and the PRCs type I, the leader neuron 1 advances its next spike by a larger amount than the follower neuron 2 (Fig. 7A). In case of excitatory neurons and type II PRCs, depending on 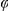 and 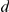, the phase of neuron 1 is advanced by a larger amount or delayed by a smaller amount than the phase of neuron 2, the latter of which is shown by the changed spike times in Fig. 7B. It is also possible that the phase of the leader neuron is advanced while that of the follower neuron is delayed. Hence, for either PRC type, 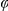 increases due to delayed excitatory coupling, that is, synchrony is destabilized. For inhibitory synapses and type I PRCs, the leader neuron 1 delays its subsequent spike by a larger amount than the follower neuron 2 (Fig. 7C). In case of type II PRCs, neuron 1 experiences a weaker phase advance or stronger phase delay than neuron 1, or else the phase of neuron 1 is delayed while that of neuron 1 is advanced, depending on 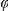 and 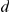 (Fig. 7D). Thus, delayed inhibitory coupling causes 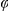 to decrease towards zero for either PRC type, that is, synchrony is stabilized.

#### Phase locking of aEIF pairs coupled with delays and unequal synaptic strengths

**Figure 8:**
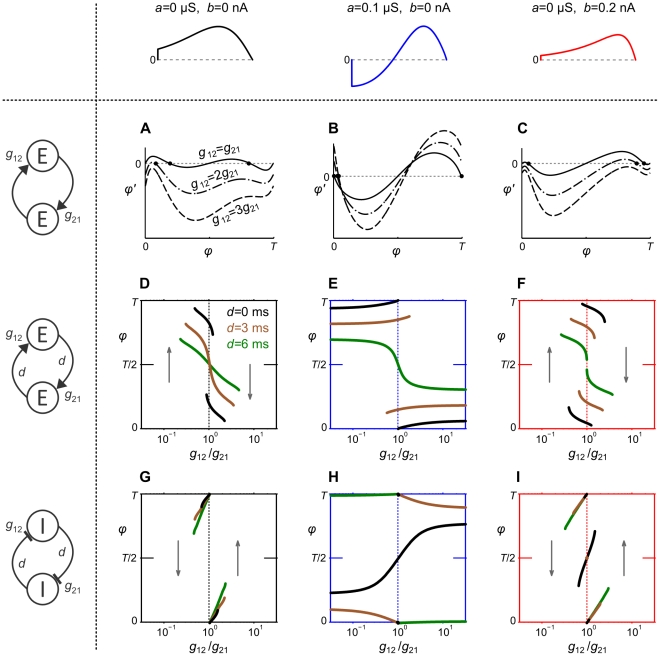
Phase locking of aEIF pairs coupled with delays and heterogeneous synaptic strengths. A–C: Change of phase difference 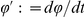 given by equation (25), as a function of 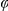 for pairs of excitatory aEIF neurons coupled with different ratios of synaptic conductances 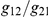 (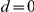). Zero crossings with a negative slope indicate stable phase locking and are marked by black dots. Adaptation parameters of the neurons and PRCs are shown in the top row. D–I: Stable phase locked states of excitatory (D–F) and inhibitory (G–I) pairs as a function of the synaptic conductance ratio, for three different conduction delays 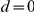, 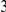 and 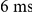 (black, brown, green). Unstable states are not shown for improved clarity. Dashed lines denote equal synaptic strengths, grey arrows indicate a continuous increase or decrease of 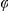 (mod 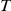) for ratios 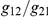 at which phase locked states do not exist (see main text).

In the following we analyze phase locking of neuronal pairs with unequal synaptic peak conductances 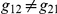. Due to the linearity of the integral in eq. (21) we can substitute 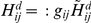 in eq. (23), which yields
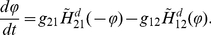
(25)By setting eq. (25) to zero, we obtain the condition eq. (26) for the existence of phase locked states,
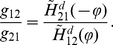
(26)Phase locked states therefore only exist if the ratio of conductances 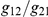 is not larger than the maximum of the periodic function 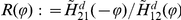. This upper bound primarily depends on the type of the PRCs and the synaptic time constants. In case of type I PRCs, 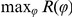 is limited because the minimum of 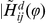 is positive. 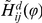 is either positive (for excitatory synapses) or negative (for inhibitory synapses) for all 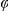. 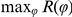 is small for slow synapses, since the slower the synaptic rise and decay times, the larger 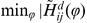, see Fig. 4A. For a type II PRC on the other hand, this minimum is zero (unless the negative lobe of the PRC is small and the synapse slow), from which follows that 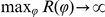. The effects of heterogeneous synaptic strengths on phase locking of neuronal pairs without adaptation, as well as either adaptation parameter increased, are shown in Fig. 8. For excitatory pairs coupled without a conduction delay it is illustrated, how the right hand side of eq. (25) changes when the coupling strengths are varied (A–C). In addition, stable phase locked states of excitatory and inhibitory pairs coupled through synapses with various mutual conduction delays (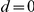, 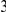, or 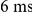) are displayed as a function of 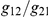 (D–I). When the ratio of conductances 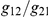 is increased, the zero crossings of 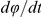 given by eq. (25), i.e. phase locked states, disappear for neurons with type I PRCs (through a SN bifurcation). 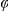 then continuously increases (or decreases) (mod 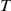) as shown by the dashed curves (without roots) in Fig. 8A,C and indicated by the arrows in Fig. 8D,F,G,I. This means, the spike frequency of one neuron becomes faster than that of the other neuron. Neurons with type II PRCs on the other hand have stable phase locked states even for diverging coupling strengths. Bistability of two phase locked states can occur for a ratio 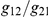 close to one (equal coupling strengths), depending on the PRC and the delay. Synchronization of excitatory-inhibitory pairs is not considered in this paper. It should be noted however, that if both neurons have type I PRCs, phase locking is not possible, irrespective of the ratio of coupling strengths. In this case, one interaction function is strictly positive and the other strictly negative and thus, the condition (26) for fixed points of eq. (25) cannot be fulfilled.

### Synchronization and clustering in aEIF networks

**Figure 9:**
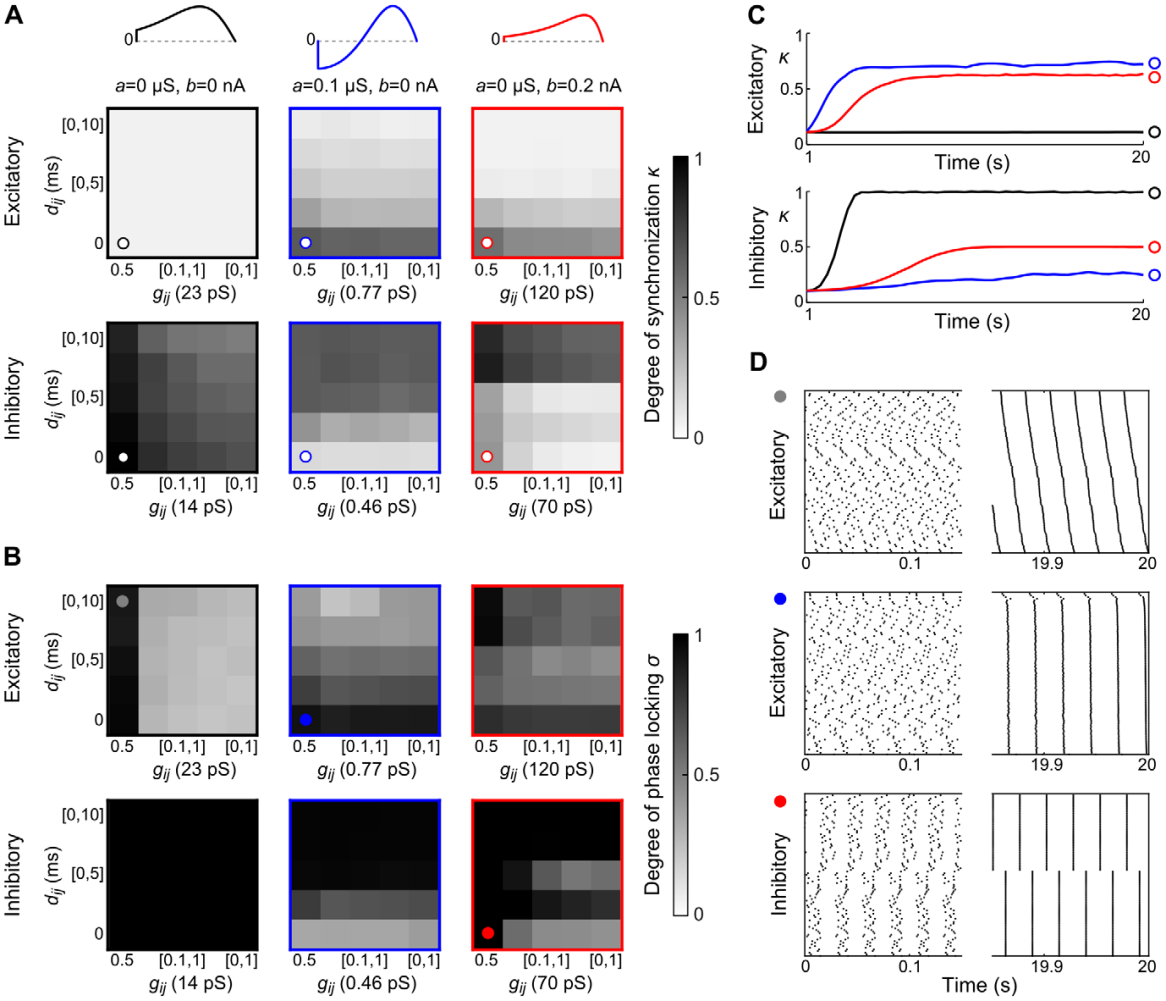
Impact of adaptation on the behavior of aEIF networks. Degree of network synchronization 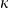 (A) and phase locking 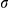 (B) of 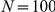 aEIF neurons without adaptation, 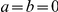 (black frame) and either adaptation component, respectively, 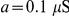, 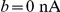 (blue frame), 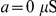, 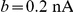 (red frame), driven to 40 Hz spiking, all-to-all coupled without self-feedback, for various conduction delays and synaptic conductances. 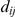 and 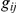 are random (uniformly distributed) in the indicated intervals. Specifically, 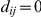, 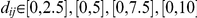 and 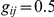, 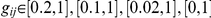, with units in parenthesis. The PRCs of the three neuron types described above are shown in the top row. C: Time course of 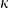 for networks without delays and equal synaptic strengths, as indicated by the symbols in A. Each 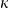 and 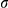 value represents an average over three simulation runs. D: Raster plots for neuron and network parameters as indicated by the symbols in B, where the neurons in the columns are sorted according to their last spike time.

In order to examine how the behavior of pairs of coupled phase neurons relates to networks of spiking neurons, we performed numerical simulations of networks of oscillating aEIF neurons without adaptation and with either a subthreshold or a spike-triggered adaptation current, respectively, and analyzed the network activity. The neurons were all either excitatory or inhibitory and weakly coupled. Fig. 9 shows the degree of synchronization 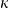 (A, C) and the degree of phase locking 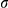 (B) for these networks considering equal as well as heterogeneous conduction delays and synaptic conductances. An increase of either adaptation parameter (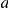 or 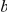) leads to increased 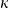 in networks of excitatory neurons with short delays. It can be recognized however, that 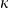 increases to larger values and this high degree of synchrony seems to be more robust against heterogeneous synaptic strengths, when the neurons are equipped with a subthreshold adaptation current (Fig. 9A,C). These effects correspond well to those of the adaptation components 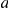 and 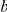 on synchronization of pairs, presented in the previous section. Parameter regimes (w.r.t. 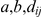 and 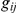) that cause stable in-phase or near in-phase locking of pairs, such as subthreshold adaptation in case of short delays or spike-triggered adaptation for short delays and coupling strength ratios close to one (Fig. 6A–C and Fig. 8D–F), lead to synchronization, indicated by large 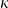 values, in the respective networks. Networks of non-adapting excitatory neurons remain asynchronous as shown by the low 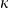 values. For equal synaptic strengths, these networks settle into splay states where the neurons are pairwise phase locked, with uniformly distributed phases (Fig. 9B,D). When the delays are large enough and the synaptic strengths equal, splay states also occur in networks of neurons with large 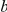, indicated by low 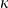 and high 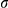 values in Fig. 9A,B. As far as inhibitory networks are concerned, non-adapting neurons synchronize, without delays or with random delays of up to 10 ms. Furthermore, synchrony in these networks is largely robust against heterogeneities in the coupling strengths (Fig. 9A). Networks of inhibitory neurons with subthreshold adaptation only show synchronization and pairwise locking for larger delays (i.e. 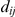 random in 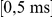 or larger). Spike-triggered adaptation promotes clustering of the network into two clusters, where the neurons within a cluster are in synchrony, as long as the delays are small. These cluster states seem to be most robust against heterogeneous synaptic strengths when the delays are small but not zero. For larger delays, inhibitory neurons of all three types (with or without adaptation) synchronize, in a robust way against unequal synaptic strengths. The behaviors of inhibitory networks are consistent with the phase locked states found in pairs of inhibitory neurons (Fig. 6D–F). Particularly, stable synchronization of pairs with larger conduction delays and the bistability of in-phase and anti-phase locking of pairs with spike-triggered adaptation for smaller delays, nicely carry over to networks. In the former case, synchrony of pairs relates to network synchrony, in the latter case, bistability of in-phase and anti-phase locking of individual pairs can explain the observed two cluster states. Note that bistability of in-phase and anti-phase locking is also shown for inhibitory pairs with subthreshold adaptation and 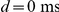. In this case however, the slope of 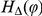 at 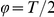 is almost zero (not shown), which might explain why the corresponding networks do not develop two-cluster states. The behavior of all simulated networks does not critically depend on the number of neurons in the network, as we obtain qualitatively similar results for network sizes changed to 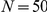 and 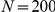 (not shown). The numerical simulations demonstrate that stable phase locked states of neural pairs can be used to predict the behavior of larger networks.

### Synchronization properties of Traub neurons with adaptation currents 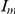, 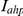


To understand the biophysical relevance of the subthreshold and spike-triggered adaptation parameters, 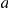 and 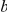, in the aEIF model, we compare them with the adaptation currents 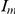 and 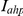 in a variant of the Hodgkin-Huxley type Traub model neuron. Specifically, in this section we investigate the effects of the low- and high-threshold currents 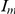 and 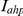, respectively, on spiking behavior, 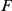-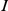 curves and PRCs of single neurons, and on synchronization of pairs and networks, using the Traub model, and compare the results with those of the previous two sections. It should be stressed, that the aEIF model was not fit to the Traub model in this study. Therefore, the comparison of how adaptation currents affect SFA, PRCs and synchronization in both models, are rather qualitative than quantitative.

#### PRC characteristics of Traub neurons

**Figure 10:**
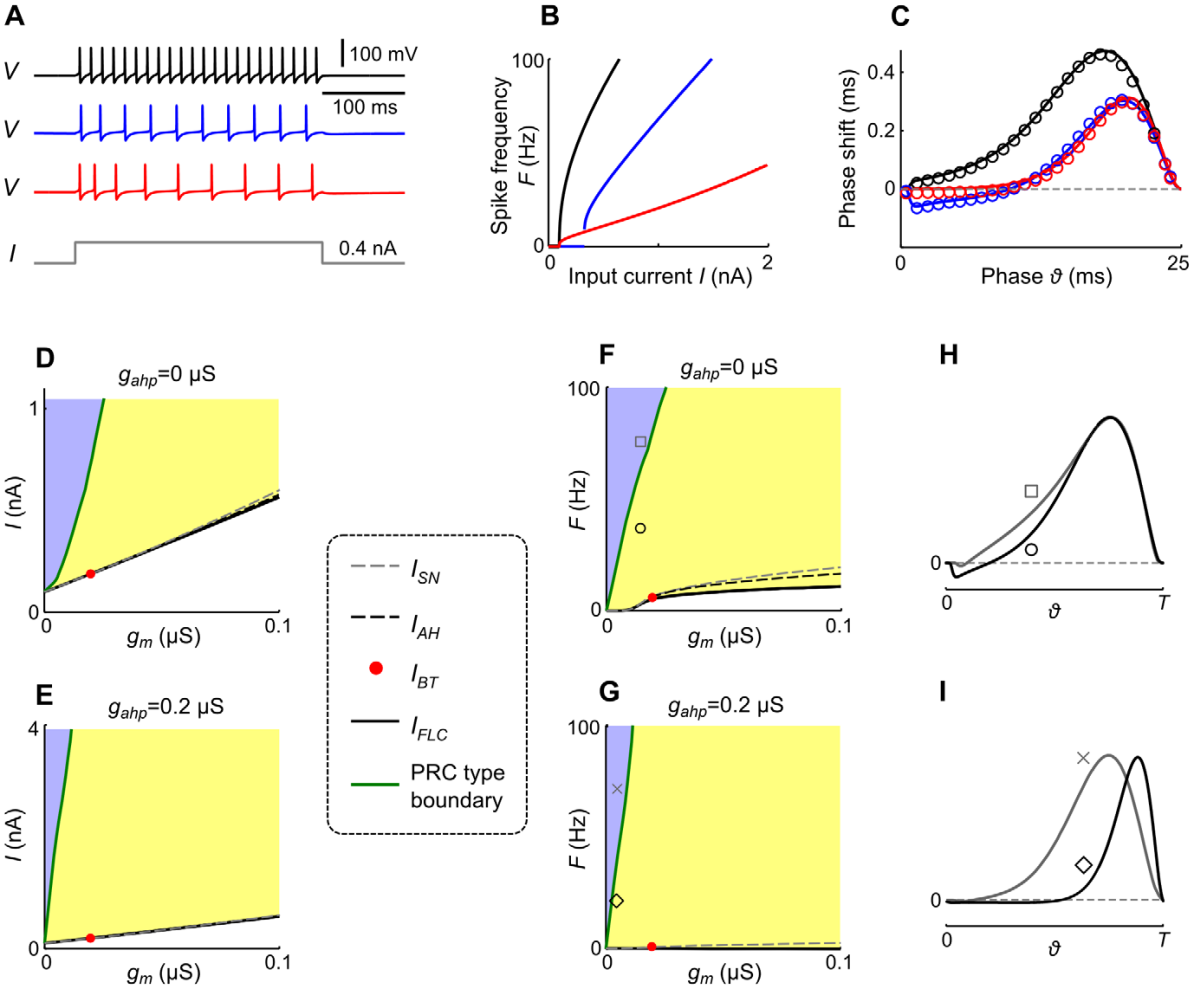
Effects of adaptation on spiking dynamics, 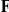-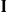 curves, PRCs and bifurcation currents of Traub model neurons. A: Membrane potential 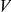 of Traub model neurons without adaptation, 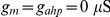 (black), 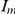-mediated, 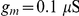 (blue) and 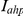-mediated adaptation, 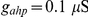 (red), in response to step currents 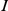, B: the corresponding 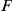-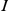 curves, and C: the corresponding PRCs. Solid lines in C denote the PRCs, calculated with the adjoint method and scaled by 0.2 mV. Open circles denote the results of numerical simulations of eqs. (4)–(9) with 0.2 mV perturbations at various phases. D,E: Rheobase current 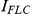 (solid black), 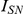 (dashed grey) and 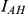 (dashed black), as a function of 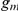, for 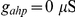 (left) and 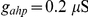 (right). 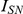 and 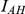 converge at 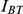 marked by the red dot. The input current indicated by the green curve separates type I and type II PRC regions (blue and yellow, respectively). F,G: Spike frequencies 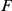 according to the input currents 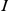 in D and E. H,I: PRCs for parametrizations as indicated in F and G (with 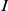 corresponding to 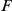), scaled to the same period 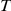. All other model parameters are provided in the Methods section.

Without adaptation, 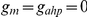 (hence 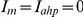), the model exhibits tonic spiking in response to a rectangular current pulse (Fig. 10A). When either adaptation current is present, that is the conductance 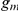 or 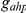 is increased to 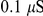, the membrane voltage trace reveals SFA. Note that 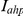 causes stronger differences in subsequent ISIs after stimulus onset, when comparing the 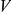-traces of neurons with either adaptation conductance set to 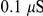. The 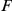-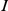 curves in Fig. 10B indicate that the presence of 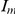 predominantly has a subtractive effect on the neuron's 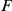-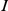 curve and gives rise to class II excitability. The presence of 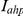 on the other hand flattens the 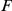-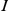 curve, in other words its effect is divisive. Furthermore, an increase of 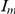 changes a type I PRC to type II, whereas increased 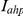 reduces its amplitude at early phases and skews its peak to the right (Fig. 10C). Evidently, the effects of 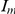 and 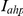 on SFA, 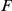-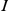 curves and PRCs of Traub neurons are consistent with the effects of the adaptation parameters 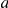 and 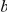 in aEIF neurons (Figs. 1, 2).

We further show how the PRC characteristics caused by the adaptation currents depend on the injected current 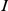, hence the spike frequency 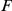, and the bifurcation type of the rest-spiking transition (Fig. 10D–I). An increase of 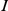 reduces the effects of 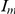 and 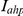 on the PRC. That means, at higher frequencies 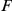, larger levels of 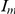 and 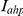 are required to obtain type II and skewed PRCs, respectively. This frequency dependence of adaptation current-mediated changes of the PRC is similar in both neuron models (Figs. 3, 10D–I). Note, that in the Traub model a rather low value of 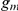 (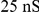) is sufficient to guarantee a type II PRC for spike frequencies of up to 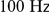 (Fig. 10F,G), compared to the aEIF model, where a much larger value of 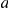 (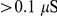) would be necessary (Fig. 3C,D). As far as the bifurcation structures of both models are concerned, an increase of the low-threshold adaptation parameters 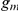 and 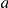 has a comparable effect in the Traub and the aEIF models, respectively, changing the transition from rest to spiking from a SN via a BT to an AH bifurcation. The exact conductance values at which this change, i.e. the BT bifurcation, occurs, differ (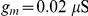 for the Traub model and 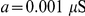 for the aEIF model).

#### Synchronization of coupled Traub neurons

**Figure 11:**
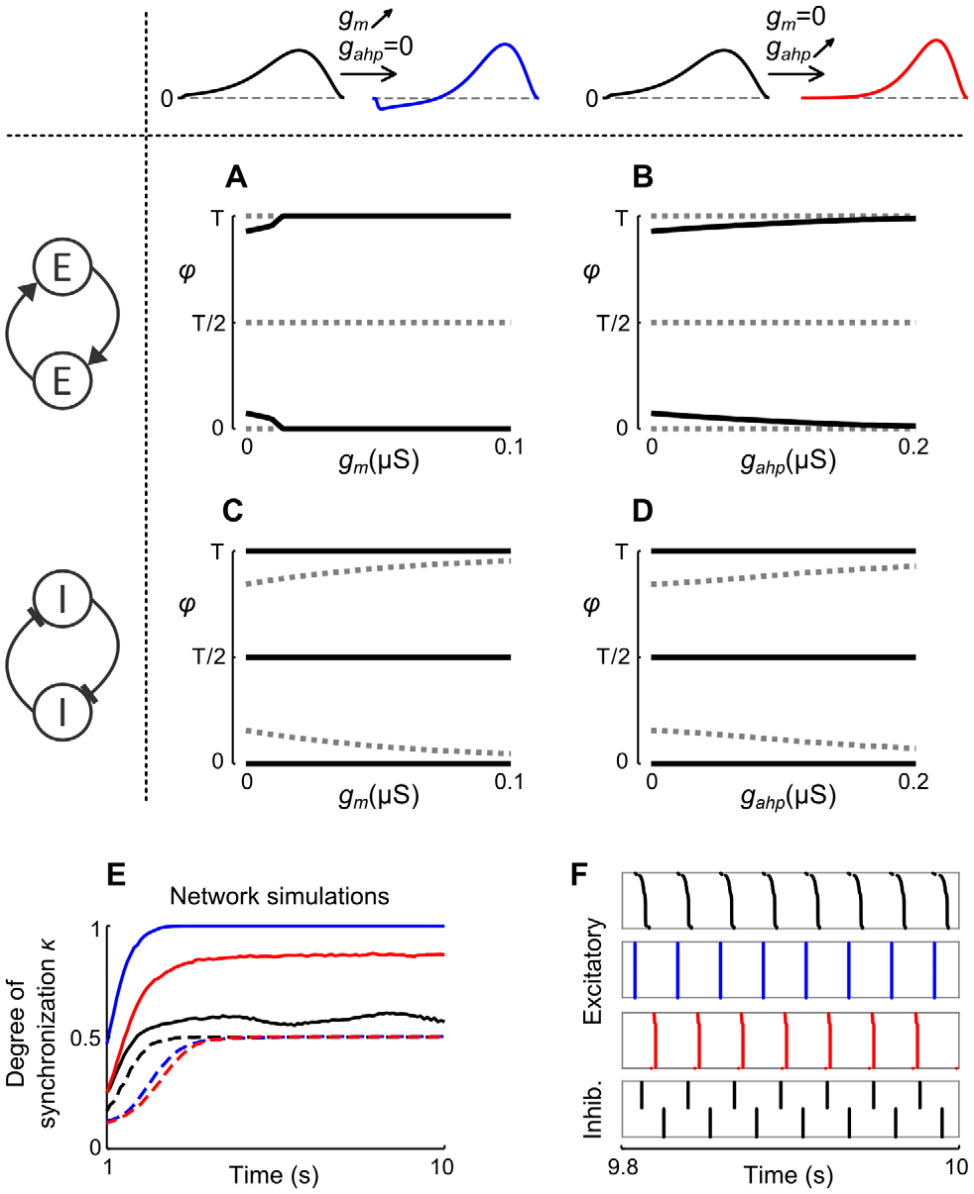
Influence of adaptation on synchronization properties of Traub model neurons. A–D: Stable (solid black) and unstable (dashed grey) phase locked states of coupled pairs of Traub neurons with identical PRCs, as a function of conductances 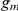 and 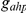, respectively. Corresponding changes in PRCs are displayed in the top row. The neurons are coupled through excitatory or inhibitory synapses as indicated by the diagrams on the left, with equal synaptic strengths, 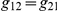 and 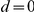. E: Network synchronization 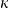 over time, of 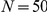 coupled excitatory (solid) and inhibitory (dashed) Traub neurons without, 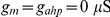 (black) or with adaptation, 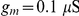, 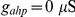 (blue) and 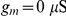, 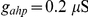 (red), driven to 40 Hz spiking. The neurons are all-to-all coupled with equal synaptic conductances, 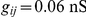 (black and blue), 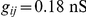 (red), but without self-feedback, 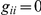, and conduction delays, 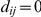. F: Raster plots showing the spike times during the last 200 ms for the three excitatory networks and the network of inhibitory neurons without adaptation (bottom). The neurons in the columns are sorted according to their last spike time.

We show the effects of the adaptation currents 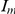 and 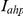 on phase locked states of pairs of Traub neurons symmetrically coupled without conduction delays in Fig. 11A–D. Excitatory pairs of neurons without adaptation phase lock with a small phase difference. Low levels of 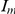 are sufficient to stabilize in-phase locking, by turning the PRC from type I to II (Fig. 11A), while an increase of 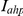 reduces the locked phase difference to almost but not exactly zero, that is, near in-phase locking, by skewing the PRC (Fig. 11B). Inhibitory synaptic coupling produces bistability of in-phase (synchrony) and anti-phase locking (anti-synchrony) for pairs of neurons without adaptation or either adaptation current increased (Fig. 11C,D). Note that the domain of attraction of the anti-synchronous state grows with increasing 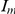 or 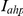, while that of the synchronous state shrinks. In contrast to the aEIF model, this bistability also occurs for neurons without an adaptation current (compare Figs. 5C,D, 11C,D).

The effects of 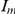 or 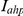 on synchronization of networks of Traub neurons coupled without conduction delays and equal synaptic strengths, are shown in Fig. 11E,F. In correspondence with the effects on pairs, 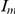 and 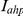 promote synchronization of excitatory networks, shown by the course of network synchronization measure 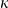 over time (Fig. 11E). The mean values of phase locking measure 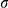 are 0.26 for nonadapting neurons and 0.98 for networks where either adaptation current is increased. An increased adaptation current 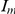 leads to larger 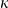 values, compared to an increase of 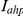, which is similar to the aEIF networks where increased 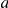 causes larger 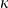 values than an increase of 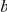 (compare Figs. 9C, 11E). In contrast to networks of excitatory aEIF neurons without adaptation, which develop splay states, 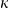 values of nonadapting excitatory Traub neuron networks increase to about 0.5, while low 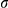 values indicate poor phase locking, hence splay states do not occur (Fig. 11F). Networks of inhibitory neurons organize into clusters, indicated by 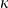 values that converge to 0.5 (Fig. 11E) and large 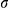 values (0.96 without adaptation, 0.94 for either 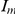 or 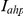 increased). Particularly, clustering into two clusters was revealed by the raster plots, see Fig. 11F. These two-cluster states of networks can be explained by the bistability of synchrony and anti-synchrony of individual pairs. Clustering emerges for all three types of Traub neurons, with and without adaptation, as opposed to networks of inhibitory aEIF neurons, where cluster states only occur in case of spike-triggered adaptation (Fig. 9). Considering the collective behavior of coupled excitatory neurons, the synchronizing effects of 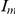 and 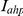 in the Traub model are comparable to those of the adaptation components 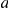 and 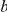 in the aEIF model.

## Discussion

In this work we studied the role of adaptation in the aEIF model as an endogenous neuronal mechanism that controls network dynamics. We described the effects of subthreshold and spike-triggered adaptation currents on the PRC in dependence of spike frequency. To provide insight into the synchronization tendencies of coupled neurons, we applied a common phase reduction technique and used the PRC to describe neuronal interaction [48], [55]. For pairs of coupled oscillating neurons we analyzed synchrony and phase locking under consideration of conduction delays and heterogeneous synaptic strengths. We then performed numerical simulations of aEIF networks to examine whether the predicted behavior of coupled pairs relates to the activity of larger networks. Finally, to express the biophysical relevance of the elementary subthreshold and spike-triggered adaptation mechanisms in the aEIF model, we compared their effects with those of the adaptation currents 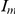 and 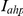 in the high-dimensional Traub neuron model, on single neuron as well as network behavior.

Conductance 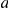, which mostly determines the amount of adaptation current in absence of spikes, that is, subthreshold, qualitatively changes the rest-spiking transition of an aEIF neuron, from a SN to an AH via a BT bifurcation as 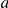 increases. Thereby the neuron's excitability, as defined by the 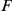-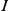 curve, and its PRC, are turned from class I to class II, and type I to type II, respectively. A similar effect of a slow outward current that acts in the subthreshold regime on the PRC has recently been shown for a two-dimensional quadratic non-leaky integrate-and-fire (QIF) model derived from a normal form of a dynamical model that undergoes a BT bifurcation [18], [48]. The relation between the PRC and the bifurcation types has further been emphasized by Brown et al. [47] who analytically determined PRCs for bifurcation normal forms and found type I and II PRC characteristics for the SN and AH bifurcations, respectively. A spike-triggered increment 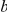 of adaptation current does not affect the bifurcation structure of the aEIF model and leaves the excitability class unchanged. When 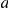 is small such that the model is in the SN bifurcation regime, an increase of 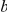 cannot change the PRC type. In the AH bifurcation regime, 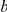 substantially affects the range of input current for which the PRC is type II but causes only a small change in the corresponding frequency range. Furthermore, spike-triggered adaptation strongly influences the skew of the PRC, shifting its peak towards the end of the ISI for larger values of 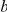. Such a right-skewed PRC implies that the neuron is most sensitive to synaptic inputs that are received just before it spikes. Similar effects of spike-triggered negative feedback with slow decay on the skew of the PRC have been reported for an extended QIF model [18], [22], [48], [58].

PRCs determine synchronization properties of coupled oscillating neurons. When the synapses are fast compared to the oscillation period, the stability of the in-phase and anti-phase locked states (which always exist for pairs of identical neurons) can be “read off” the PRC for any mutual conduction delay, as we have demonstrated. A similar stability criterion that depends on the slopes of the PRCs at the phases at which the inputs are received has recently been derived for pairs of pulse-coupled oscillators [59]. Under the assumption of pulsatile coupling, the effect of a synaptic input is required to dissipate before the next input is received. In principle, the synaptic current can be strong, but it must be brief such that the perturbed trajectory returns to the limit cycle before the next perturbation occurs [14].

We have shown that, as long as synaptic delays are negligible and synaptic strengths equal, excitatory pairs synchronize if their PRCs are type II, as caused by 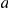, and lock almost in-phase if their PRCs are type I with a strong skew, as mediated by 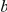. Inhibitory pairs synchronize in presence of conduction delays and show bistability of in-phase and anti-phase locking for small delays, particularly in case of skewed PRCs. Conduction delays and synaptic time constants can affect the stability of synchrony in a similar way, by producing a lateral shift of the interaction function 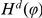, as shown in Fig. 4. Note however, that the synaptic timescale has an additional effect on the shape of 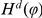, smoothing it for slower synaptic rise and decay times. We have further demonstrated that heterogeneity in synaptic strengths desynchronizes excitatory and inhibitory pairs and leads to phase locking with a small phase difference in case of type II PRCs and small delays. While neurons with type II PRCs have stable phase locked states even for large differences in synaptic strengths, pairs of coupled neurons with type I PRCs are only guaranteed to phase lock when the synaptic strengths are equal. Similar effects of heterogeneous synaptic conductances have recently been observed in a computational study of weakly coupled Wang-Buszaki and Hodgkin-Huxley neurons (with class I and II excitability, respectively) [60].

The activity of larger aEIF networks, simulated numerically, is consistent with the predictions of the behavior of pairs. In fact, knowledge on phase locking of coupled pairs helps to explain the observed network states. Both adaptation mediated PRC characteristics, i.e. a negative lobe or a pronounced right skew, favor synchronization in networks of excitatory neurons, in agreement with previous findings [17], [22], [61]. This phenomenon only occurs when the conduction delays are negligible. It has been shown previously that synchrony in networks of excitatory oscillators becomes unstable when considering coupling with delays [62], [63]. We have demonstrated that increased conduction delays promote asynchrony in excitatory networks, with or without adaptation currents. Inhibitory neurons on the other hand are able to synchronize spiking in larger networks for a range of conduction delays. This provides support to the hypothesis that inhibitory networks play an essential role in generating coherent brain rhythms, as has been proposed earlier [43], [64], [2] for review. Inhibition rather than excitation has been found to generate neuronal synchrony particularly in case of slow synaptic rise and decay [40], [61], [65], and in the presence of conduction delays as has recently been shown experimentally [66]. In regimes that lead to bistability of in-phase and anti-phase locking according to our analysis of pairs, the simulated networks break up into two clusters of synchronized neurons. Recently it has been shown that a stable two cluster state of pulse coupled neural oscillators can exist even when synchrony of individual pairs is unstable [67]. Such cluster states have been invoked to explain population rhythms measured in vitro, where the involved neurons spike at about half of the population frequency [68].

Spike frequency has been shown to affect the skewness of PRCs, using type I integrate-and-fire neurons with adaptation [58], and to modulate the negative lobe in type II PRCs of conductance based model neurons [45]. Using the aEIF model we have demonstrated that the spike frequency strongly attenuates the effect of either adaptation mechanism on the PRC. At high frequency, unphysiologically large adaptation parameter values are necessary to produce a negative lobe or a significant right-skew in the PRC. This means, for a given degree of adaptation in excitatory neurons, synchronization is possible at frequencies up to a certain value. The stronger the adaptation, the larger this upper frequency limit. It has been previously suggested that the degree of adaptation can determine a preferred frequency range for synchronization of excitatory neurons, based on the observation (in vitro and in silico) that the neurons tend to spike in phase with injected currents oscillating at certain frequencies [69]. This preferred oscillation frequency increases with increasing degree of SFA. According to our results, at low frequencies synchronization of local circuits through excitatory synapses is possible, provided that the neurons are adapting and delays are short. At higher frequencies, adaptation much less affects the synchronization tendency of excitatory neurons and inhibition may play the dominant role in generating coherent rhythms [43], [64].

The adaptation currents 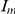 and 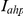 have previously been found to influence the phase response characteristics of the biophysical Traub neuron model, turning a type I PRC to type II (through 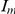) and modulating its skew (through 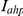) [18], [22]. We have shown that these changes of the PRC are reflected in the aEIF model by its two adaptation parameters and that in both models (aEIF and Traub) these changes are modulated by the spike frequency. As a consequence, the adaptation induced effects on synchronization of pairs and networks of oscillating neurons are qualitatively similar in both models. Quantitative differences with respect to these effects may well be reduced by fitting the aEIF model parameters to Traub neuron features.

Our analysis of phase locked states is based on the assumption that synaptic interactions are weak. Experimental work lending support to this assumption has been reviewed in [14], [50]. Particularly for stellate cells of the entorhinal cortex, synaptic coupling has been found to be weak [70]. Another assumption in this study is that the neurons spike with the same frequency. Considering a pair of neurons spiking at different frequencies, equation (23) needs to be augmented by a scalar 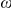, which accounts for the constant frequency mismatch between the two neurons [71]: 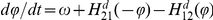. In this case, the condition for the existence of phase locked states is 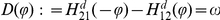. Due to the assumption of weak synaptic strengths however, 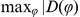 must be small, which means that the above condition can only be met if 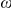 is small. In other words, in the limit of weak coupling phase locking is only possible if the spike frequencies are identical or differ only slightly. The phase reduction technique considered here, and PRCs in general, are of limited applicability for studying network dynamics in a regime where individual neurons spike at different frequencies, or even irregularly. How adaptation currents affect network synchronization and rhythm in such a regime nevertheless remains an interesting question to be addressed in the future.

## Supporting Information

Text S1 Supplementary Methods. A) Calculation of the PRC using the adjoint method. B) Phase reduction.
(PDF)
