# Supplementary material for: Impact of Adaptation Currents on Synchronization of Coupled Exponential Integrate-and-Fire Neurons
Source: PLoS Comput Biol. 2012 Apr 12;8(4):e1002478. doi: 10.1371/journal.pcbi.1002478 (PMC3325187; doi:10.1371/journal.pcbi.1002478)
Supplement: Text S1 — Supplementary Methods. A) Calculation of the PRC using the adjoint method. B) Phase reduction. (PDF) [file pcbi.1002478.s001.pdf]

Impact of adaptation currents on synchronization of coupled exponential integrate-and-fire neurons

Josef Ladenbauer[1,2*], Moritz Augustin[1], LieJune Shiau[3], Klaus Obermayer[1,2]
1 Department of Software Engineering and Theoretical Computer Science, Technische Universität Berlin, Berlin, Germany
2 Bernstein Center for Computational Neuroscience Berlin, Berlin, Germany
3 Department of Mathematics, University of Houston, Houston, Texas, United States of America
∗ **E-mail: jl@ni.tu-berlin.de**

Text S1 - Supplementary Methods

A) Calculation of the PRC using the adjoint method

Let $\mathbf{x} \in \mathbb{R}^n$, $\mathbf{f} : \mathbb{R}^n \to \mathbb{R}^n$, and let $\bar{\mathbf{x}}(t)$ be the T-periodic asymptotically stable spiking trajectory as a solution of the system of differential equations

$$\frac{d\mathbf{x}}{dt} = \mathbf{f}(\mathbf{x}), \tag{27}$$

which describes an uncoupled neuron (cf. Methods). In case of the aEIF model, eq. (27) is extended by a reset condition, leading to discontinuities of $\bar{\mathbf{x}}(t)$ at $t \neq kT,\ k \in \mathbb{Z}$. We define the phase $\vartheta \in [0, T)$ of $\bar{\mathbf{x}}(t)$, by a differentiable 1:1-mapping Θ between the points on the periodic spiking trajectory $\{\bar{\mathbf{x}}(t) : t \in \mathbb{R}\}$ and the interval $[0, T)$, $\Theta(\bar{\mathbf{x}}(\vartheta)) = \vartheta$, where $\vartheta = 0$ corresponds to the spike time. Next, we extend the domain of Θ to points in the neighborhood of $\bar{\mathbf{x}}(t)$. Suppose $\mathbf{x}_0$ is a point on the trajectory $\bar{\mathbf{x}}(t)$, $\mathbf{y}_0$ is a point within its domain of attraction, and $\mathbf{x}(t)$, $\mathbf{y}(t)$ are the solutions of eq. (27) plus the reset condition in case of the aEIF model with initial conditions $\mathbf{x}_0$, $\mathbf{y}_0$. The phase of $\mathbf{y}_0$, $\Theta(\mathbf{y}_0)$, is then defined by $\Theta(\mathbf{x}_0) = \Theta(\mathbf{y}_0)$ if $\lim_{t\to\infty} ||\mathbf{x}(t) - \mathbf{y}(t)|| = 0$.

Let $\mathbf{p} \in \mathbb{R}^n$ be a small perturbation at phase ϑ which changes the phase of the neuron to ϑ_{pert}. This changes the time of the next spike to $T_{pert} = \vartheta + T - \vartheta_{pert}$. We then obtain for the PRC

$$\mathrm{PRC}(\vartheta) = T - T_{pert}(\vartheta) = \vartheta_{pert} - \vartheta = \Theta(\bar{\mathbf{x}}(\vartheta) + \mathbf{p}) - \Theta(\bar{\mathbf{x}}(\vartheta)) = \nabla\Theta(\bar{\mathbf{x}}(\vartheta))^T\mathbf{p} + O(||\mathbf{p}||^2), \tag{28}$$

where we have applied Taylor expansion of $\Theta(\bar{\mathbf{x}}(\vartheta) + \mathbf{p})$ around $\bar{\mathbf{x}}(\vartheta)$. As $\Theta(\bar{\mathbf{x}}(t) + \mathbf{p})$ is rather difficult to calculate, we instead compute $\nabla\Theta(\bar{\mathbf{x}}(t))$, as explained in the following.

Let $\bar{\mathbf{x}}(t) + \mathbf{z}(t)$ be a solution of eq. (27) with initial condition $\bar{\mathbf{x}}(\vartheta) + \mathbf{z}(\vartheta) = \bar{\mathbf{x}}(\vartheta) + \mathbf{p}$ close to the periodic spiking trajectory, i.e. $\mathbf{z}(t)$ is the deviation from $\bar{\mathbf{x}}(t)$ for $t \geq \vartheta$. According to the definition of the phase function Θ, the difference of the perturbed trajectory's phase and that of the periodic attractor $\bar{\mathbf{x}}(t)$ is independent of time, that is

$$\Theta(\bar{\mathbf{x}}(t) + \mathbf{z}(t)) - \Theta(\bar{\mathbf{x}}(t)) = c \in \mathbb{R} \quad \forall t \geq \vartheta, \tag{29}$$

which can be rewritten as $\nabla\Theta(\bar{\mathbf{x}}(t))^T\mathbf{z}(t) + O(||\mathbf{z}(t)||^2) = c$ using Taylor expansion, since Θ is differentiable. We neglect terms of second and higher order and define $\mathbf{q}(t) := \nabla\Theta(\bar{\mathbf{x}}(t))$ to obtain

$$\mathbf{q}(t)^T\mathbf{z}(t) = c \quad \forall t \geq \vartheta. \tag{30}$$

For $\vartheta < t < T$, eq. (30) implies

$$\frac{d}{dt}\big(\mathbf{q}(t)^T\mathbf{z}(t)\big) = \frac{d\mathbf{q}(t)^T}{dt}\mathbf{z}(t) + \mathbf{q}(t)^T D_{\mathbf{x}}\mathbf{f}(\bar{\mathbf{x}}(t))\mathbf{z}(t) = \left(\frac{d\mathbf{q}(t)^T}{dt} + D_{\mathbf{x}}\mathbf{f}(\bar{\mathbf{x}}(t))^T\mathbf{q}(t)\right)^T \mathbf{z}(t) = 0, \tag{31}$$

where we have used the chain rule and the fact that $\mathbf{z}(t)$ satisfies the variational equation

$$\frac{d\mathbf{z}(t)}{dt} = D_{\mathbf{x}}\mathbf{f}(\bar{\mathbf{x}}(t))\mathbf{z}(t) \tag{32}$$

up to an error of $O(||\mathbf{z}(t)||^2)$, which can be neglected. Since $\mathbf{p}$ and thus $\mathbf{z}(t)$ are arbitrary, $\mathbf{q}(t)$ satisfies the linearized adjoint equation

$$\frac{d\mathbf{q}(t)^T}{dt} = -D_{\mathbf{x}}\mathbf{f}(\bar{\mathbf{x}}(t))^T\mathbf{q}(t). \tag{33}$$

In case of the aEIF model we have a discontinuity in $\bar{\mathbf{x}}(t)$ for $t = T$. At this point, the displacement $\mathbf{z}(t)$ of $\bar{\mathbf{x}}(t)$ changes discontinuously according to $\mathbf{z}(T) = A\mathbf{z}(T^-)$, where

$$A = \left(\frac{d\bar{V}}{dt}(T^-)\right)^{-1}\begin{pmatrix} \frac{d\bar{V}}{dt}(0) & 0 \\ \frac{d\bar{w}}{dt}(0) - \frac{d\bar{w}}{dt}(T^-) & \frac{d\bar{V}}{dt}(T^-) \end{pmatrix}. \tag{34}$$

A derivation is provided in [1]. The corresponding transition for the adjoint, $\mathbf{q}(T) = B\mathbf{q}(T^-)$ can be obtained using eq. (30),

$$\mathbf{q}(T)^T\mathbf{z}(T) = \mathbf{q}(T)^T A\mathbf{z}(T^-) = \left(B\mathbf{q}(T^-)\right)^T A\mathbf{z}(T^-) = \mathbf{q}(T^-)^T B^T A\mathbf{z}(T^-) = \mathbf{q}(T^-)^T\mathbf{z}(T^-). \tag{35}$$

That is, matrix B, which accounts for the jump of $\mathbf{q}(t)$ is given by $B = A^{-T}$, see e.g. [2,3]. Note that for a continuous neuron model such as the Traub model, $A = B = I$, where I is the identity matrix.

For $T < \vartheta \leq T + \vartheta$, $\mathbf{q}(t)$ satisfies the linearized adjoint eq. (33), which follows again from eq. (30). As $\mathbf{q}(t)$ is T-periodic, it solves eq. (33) for $t \neq kT$, $k \in \mathbb{Z}$, and the transition at $t = kT$ is given by $\mathbf{q}(kT) = B\mathbf{q}(kT^-)$. By differentiating $\Theta(\bar{\mathbf{x}}(\vartheta)) = \vartheta$ with respect to ϑ, we obtain

$$\mathbf{q}(\vartheta)^T\frac{d\bar{\mathbf{x}}(\vartheta)}{d\vartheta} = \mathbf{q}(\vartheta)^T\mathbf{f}(\bar{\mathbf{x}}(\vartheta)) = 1 \quad \forall\vartheta \in (0,T), \text{ and} \tag{36}$$

$$\mathbf{q}(t)^T\mathbf{f}(\bar{\mathbf{x}}(t)) = 1 \quad \forall t \in \mathbb{R}, \tag{37}$$

using eq. (35), the T-periodicity of $\mathbf{q}(t)$, and the fact that $\mathbf{f}(\bar{\mathbf{x}})$ solves the variational eq. (32) with transition $\mathbf{f}(\bar{\mathbf{x}}(kT)) = A\mathbf{f}(\bar{\mathbf{x}}(kT^-))$. We applied eq. (37) as a normalization condition to determine the appropriate solution of the adjoint system as explained below.

Any T-periodic $\tilde{\mathbf{q}}(t)$ that solves the adjoint system eq. (33) for $t \neq kT$ and fulfills $\tilde{\mathbf{q}}(kT) = B\tilde{\mathbf{q}}(kT^-)$ for $t = kT$, can be written as $\tilde{\mathbf{q}}(t) = \alpha\,\mathbf{q}(t)$, $\alpha \in \mathbb{R}$. This follows from the asymptotic stability of $\bar{\mathbf{x}}(t)$, which implies that T-periodic solutions of the variational equation (32) with transition $\mathbf{z}(kT) = A\mathbf{z}(kT^-)$ at the discontinuities, are multiples of $\mathbf{f}(\bar{\mathbf{x}}(t))$. Thus the space of T-periodic solutions of the adjoint system is one-dimensional [3,4]. The factor α can be determined by requiring that $\tilde{\mathbf{q}}(t)$ satisfies the normalization condition eq. (37) for one t. This implies that $\tilde{\mathbf{q}}(t)$ fulfills eq. (37) for all $t \in \mathbb{R}$, as can be seen from

$$\frac{d}{dt}\left(\tilde{\mathbf{q}}(t)^T\mathbf{f}(\bar{\mathbf{x}}(t))\right) = -(D_{\mathbf{x}}\mathbf{f}(\bar{\mathbf{x}}(t))^T\tilde{\mathbf{q}})^T\mathbf{f}(\bar{\mathbf{x}}(t)) + \tilde{\mathbf{q}}(t)^T D_{\mathbf{x}}\mathbf{f}(\bar{\mathbf{x}}(t))\mathbf{f}(\bar{\mathbf{x}}(t)) = 0 \tag{38}$$

for $t \neq kT$ and $\mathbf{q}(kT)^T\mathbf{f}(\bar{\mathbf{x}}(kT) = \mathbf{q}(kT^-)^T\mathbf{f}(\bar{\mathbf{x}}(kT^-)$.

In case of the continuous Traub model, we solve the linearized adjoint eq. (33) numerically backwards in time over several cycles with initial value $\mathbf{q}(0) = \mathbf{f}(\bar{\mathbf{x}}(0))$ to obtain a T-periodic solution $\tilde{\mathbf{q}}(t) = \alpha\,\mathbf{q}(t)$, and apply the normalization condition eq. (37) at $t = 0$ to fix α, i.e. $\alpha = \tilde{\mathbf{q}}(0)^T\mathbf{f}(\bar{\mathbf{x}}(0))$. For details, see e.g. [4].

In case of the aEIF model, $\mathbf{q}(t)$ is the unique solution to the linearized adjoint eq. (33), subject to the normalization condition eq. (37) at $t = 0$,

$$\mathbf{q}(0)^T\mathbf{f}(\bar{\mathbf{x}}(0)) = 1, \tag{39}$$

and the condition

$$\mathbf{q}(0) = B\mathbf{q}(T^-) \tag{40}$$

$$\text{with } B = \left(\frac{d\bar{V}}{dt}(0)\right)^{-1} \begin{pmatrix} \frac{d\bar{V}}{dt}(T^-) & \frac{d\bar{w}}{dt}(T^-) - \frac{d\bar{w}}{dt}(0) \\ 0 & \frac{d\bar{V}}{dt}(0) \end{pmatrix}, \tag{41}$$

which takes account of the discontinuity and guarantees that $\mathbf{q}(t)$ is T-periodic. One of the two scalar equations of the latter condition eq. (40) can be omitted as explained below. Eq. (39) implies that the normalization condition, eq. (37), is satisfied for all $t \in [0, T)$ (cf. eq. (38)), including $t = T^-$. We then obtain

$$\mathbf{q}(0)^T\mathbf{f}(\bar{\mathbf{x}}(0)) = \mathbf{q}(T^-)^T\mathbf{f}(\bar{\mathbf{x}}(T^-)) \tag{42}$$

$$\Longleftrightarrow q^V(0)\frac{d\bar{V}}{dt}(0) + q^w(0)\frac{d\bar{w}}{dt}(0) = q^V(T^-)\frac{d\bar{V}}{dt}(T^-) + q^w(T^-)\frac{d\bar{w}}{dt}(T^-) \tag{43}$$

$$\Longleftrightarrow q^V(0)\frac{d\bar{V}}{dt}(0) = \frac{d\bar{V}}{dt}(T^-)q^V(T^-) + \left(\frac{d\bar{w}}{dt}(T^-) - \frac{d\bar{w}}{dt}(0)\right)q^w(T^-), \tag{44}$$

where eq. (44) is the first scalar equation of eq. (40) multiplied with $\frac{d\bar{V}}{dt}(0)$. In eq. (44) we have used the second scalar equation of eq. (40),

$$q^w(0) = q^w(T^-). \tag{45}$$

It follows that if eqs. (39) and (45) are satisfied, eq. (44) and thus the first scalar equation of eq. (40) hold as well. It is therefore sufficient to solve eq. (33) for $t \in (0, T)$ using conditions (39) and (45). This is equivalent the boundary value problem, eqs. (17)–(20) from the Methods section of the main paper.

As the synaptic current only perturbs the membrane potential, the perturbation $\mathbf{p} = (p_1, 0, \ldots, 0)^T$ considered here is nonzero only in the first component. Thus, the PRC reduces to $q^V(\vartheta)\, p_1$, where q^V denotes the first component of $\mathbf{q}$. Since p_1 is only a scaling factor, in this study we identify q^V with the PRC.

B) Phase reduction

In the following we describe how the full network model eq. (10) is reduced to a lower dimensional network model where each neuron is represented by its phase ϑ_i. This phase reduction requires weak coupling between each pair of neurons which we emphasize by rewriting $I_{syn}(V_i, V_j) = \varepsilon\tilde{I}_{syn}(V_i, V_j)$, where I_{syn} is the synaptic current introduced in eq. (11) and $\varepsilon > 0$ is small (due to small conductance g_{ij}). By applying a change of variables $\vartheta_i := \Theta(\mathbf{x}_i)$ in eq. (10), with phase function Θ as defined in the previous section, the network equation for neuron i becomes

$$\frac{d\vartheta_i}{dt} = \frac{d\Theta(\mathbf{x}_i)}{dt} = \nabla\Theta(\mathbf{x}_i)^T\frac{d\mathbf{x}_i}{dt} \tag{46}$$

$$= \nabla\Theta(\mathbf{x}_i)^T\mathbf{f}(\mathbf{x}_i) + \nabla\Theta(\mathbf{x}_i)^T\sum_{j=1}^{N}\mathbf{h}_{ij}(\mathbf{x}_i, \mathbf{x}_j) \tag{47}$$

$$= 1 + \varepsilon\frac{\partial\Theta(\mathbf{x}_i)}{\partial x_1}\sum_{j=1}^{N}\tilde{I}_{syn}(\mathbf{x}_i, \mathbf{x}_j). \tag{48}$$

In eqs. (46)–(48) we have used the chain rule, the relation $\nabla\Theta(\mathbf{x}_i)^T\mathbf{f}(\mathbf{x}_i) = 1$ which is evident when considering the uncoupled system, and the fact that the coupling function $\mathbf{h}_{ij}(\mathbf{x}_i, \mathbf{x}_j)$ is nonzero only in the first component where it consists of $\varepsilon\tilde{I}_{syn}(\mathbf{x}_i, \mathbf{x}_j)$. Next, to get rid of the state variables $\mathbf{x}_i$ in eq. (48), we first approximate $\mathbf{x}_i$ using the periodic spiking trajectories parametrized by the phase $\bar{\mathbf{x}}_i(\vartheta_i)$. This approximation causes an error of $O(\varepsilon)$, which becomes $O(\varepsilon^2)$ due to the factor ε,

$$\frac{d\vartheta_i}{dt} = 1 + \varepsilon \frac{\partial\Theta(\bar{\mathbf{x}}_i(\vartheta_i))}{\partial x_1} \sum_{j=1}^{N} \tilde{I}_{syn}(\bar{\mathbf{x}}_i(\vartheta_i), \bar{\mathbf{x}}_j(\vartheta_j)) + O(\varepsilon^2). \tag{49}$$

We neglect second order terms in ε and apply another change of variables $\psi_i := \vartheta_i - t$,

$$\frac{d\psi_i}{dt} = \varepsilon \frac{\partial\Theta(\bar{\mathbf{x}}_i(t+\psi_i))}{\partial x_1} \sum_{j=1}^{N} \tilde{I}_{syn}(\bar{\mathbf{x}}_i(t+\psi_i), \bar{\mathbf{x}}_j(t+\psi_j)), \tag{50}$$

to obtain an equation to which we can apply the method of averaging, see e.g. [5], that leads to

$$\frac{d\bar{\psi}_i}{dt} = \varepsilon \frac{1}{T}\int_0^T \frac{\partial\Theta(\bar{\mathbf{x}}_i(s+\bar{\psi}_i))}{\partial x_1} \sum_{j=1}^{N} \tilde{I}_{syn}(\bar{\mathbf{x}}_i(s+\bar{\psi}_i), \bar{\mathbf{x}}_j(s+\bar{\psi}_j))ds \tag{51}$$

$$= \varepsilon \sum_{j=1}^{N} \frac{1}{T}\int_0^T \frac{\partial\Theta(\bar{\mathbf{x}}_i(s))}{\partial x_1} \tilde{I}_{syn}(\bar{\mathbf{x}}_i(s), \bar{\mathbf{x}}_j(s+\bar{\psi}_j-\bar{\psi}_i))ds, \tag{52}$$

where we have used in eq. (52) that the spiking trajectories are T-periodic. Changing the variables one more time $\bar{\vartheta}_i := t + \bar{\psi}_i$ we arrive at

$$\frac{d\bar{\vartheta}_i}{dt} = 1 + \varepsilon \sum_{j=1}^{N} \frac{1}{T}\int_0^T \frac{\partial\Theta(\bar{\mathbf{x}}_i(s))}{\partial x_1} \tilde{I}_{syn}(\bar{\mathbf{x}}_i(s), \bar{\mathbf{x}}_j(s+\bar{\vartheta}_j-\bar{\vartheta}_i))ds, \tag{53}$$

which is identical to eq. (21), recognizing that $\partial\Theta(\bar{\mathbf{x}}_i(s))/\partial x_1 = q_i^V(s)$ and $\varepsilon\tilde{I}_{syn}(V_i, V_j) = I_{syn}(V_i, V_j)$. Note that the phases ϑ_i in eqs. (21) and (22) are averaged phases $\bar{\vartheta}_i$.

References

1. Mueller PC (1995) Calculation of Lyapunov exponents for dynamic systems with discontinuities. Chaos Soliton Fract 5: 1671–1681.

2. Samoilenko AM, Perestyuk NA (1995) Impulsive differential equations. Singapore: World Scientific. 462 p.

3. Akhmet M (2010) Principles of discontinuous dynamical systems. New York: Springer. 180 p.

4. Ermentrout GB, Terman D (2010) Mathematical foundations of neuroscience. New York: Springer. 422 p.

5. Hoppenstaedt F, Izhikevich EM (1997) Weakly connected neural networks. New York: Springer. 400 p.
